# Novel Adipokines in Critical Illness and Sepsis: Chemerin, Vaspin, and Omentin-1: A Comprehensive Evidence-Based Review

**DOI:** 10.3390/biomedicines14071553

**Published:** 2026-07-10

**Authors:** Vassiliki Giannopoulou, Kostas A. Papavassiliou, Nikolaos S. Lotsios, Matina Kardara, Anastasia Kotanidou, Athanasios G. Papavassiliou, Ioanna Dimopoulou, Alice G. Vassiliou

**Affiliations:** 1 First Department of Critical Care Medicine, “Evangelismos” Hospital, Medical School, National and Kapodistrian University of Athens, 10676 Athens, Greece; vaso.giannop88@gmail.com (V.G.); n.lotsios96@gmail.com (N.S.L.); kardara.matina@gmail.com (M.K.); akotanid@med.uoa.gr (A.K.); idimop@med.uoa.gr (I.D.); 2 First University Department of Respiratory Medicine, “Sotiria” Chest Hospital, Medical School, National and Kapodistrian University of Athens, 11527 Athens, Greece; kpapavassiliou@gmail.com; 3 Department of Biological Chemistry, Medical School, National and Kapodistrian University of Athens, 11527 Athens, Greece; papavas@med.uoa.gr

**Keywords:** adipokines, chemerin, vaspin, omentin-1, sepsis, critical illness, biomarkers, in vitro models, in vivo models, inflammation

## Abstract

Adipose tissue has emerged as a pivotal endocrine organ, secreting bioactive proteins termed adipokines that regulate metabolic and immune processes across multiple organ systems. In the context of sepsis and critical illness, conditions defined by a dysregulated host response to infection with life-threatening organ dysfunction, the role of novel adipokines has attracted considerable research interest. This review focuses on three novel adipokines: chemerin, vaspin (*SERPINA12*), and omentin-1 (intelectin-1). We will discuss current in vitro, in vivo experimental animal models, and clinical evidence, emphasizing their biology, mechanisms of action, and potential as diagnostic and prognostic biomarkers in critically ill patients. All three adipokines are elevated in sepsis compared with healthy controls and correlate with established severity scores, including APACHE II and SOFA. Chemerin and omentin-1 have both been independently associated with 28-day mortality in prospective cohort studies. Vaspin exhibits robust cardioprotective effects in murine sepsis models via inhibition of kallikrein 7 (KLK7) and attenuates lipopolysaccharide (LPS)-induced acute lung injury (ALI) both in vitro and in vivo. Omentin-1 suppresses LPS-induced macrophage activation through TLR4/MyD88/NF-κB inhibition in vitro and protects against LPS-induced ALI in murine models. Despite these promising findings, substantial methodological heterogeneity and limited large-scale clinical data currently preclude clinical implementation. Future research that standardizes assays, expands to multicenter cohorts, and investigates therapeutic modulation of these pathways is urgently needed.

## 1. Introduction

Sepsis, defined by the Sepsis-3 consensus, is a life-threatening organ dysfunction caused by a dysregulated host response to infection [1]. It remains one of the foremost challenges in modern critical care medicine, with high mortality and no specific pharmacological treatments beyond supportive care. Global epidemiology data suggest an incidence of approximately 300 cases per 100,000 person-years for sepsis, with ICU mortality ranging between 22 and 35% across diverse patient populations [2]. Despite decades of research targeting the immune-inflammatory cascade, therapeutic trials have largely failed, prompting a search for novel pathophysiological mediators and biomarkers that may offer new diagnostic, prognostic, or therapeutic avenues [3,4].

White adipose tissue (WAT), long regarded solely as a passive energy store, is now established as an active endocrine and immunomodulatory organ secreting a family of bioactive proteins, collectively termed adipokines. These regulate energy homeostasis, insulin sensitivity, angiogenesis, and immune responses [5,6,7]. During critical illness, adipose tissue undergoes reprogramming, morphologically (increased adipogenesis, smaller adipocytes), immunologically (M2-macrophage accumulation), and metabolically (enhanced glucose and triglyceride storage, WAT browning), transforming into an immunometabolic hub that actively participates in the host response [5,8,9]. Du et al. conceptualized this as the “adipose–immune–metabolic axis” governing disease tolerance in sepsis through metabolic defense priority, bidirectional immunometabolic crosstalk, and stage-specific adaptation [10].

Observational studies suggest improved survival in sepsis in overweight and moderately obese critically ill patients compared to normal-weight patients, the so-called “obesity paradox” [11,12]. While multiple mechanisms have been proposed (nutritional reserves, endotoxin sequestration via lipoproteins, hemodynamic advantages from RAS activation), one of the most compelling explanations involves the secretion of anti-inflammatory adipokines by adipose tissue [12,13]. However, studies of classical adipokines (leptin, adiponectin) in relation to the obesity paradox have yielded inconclusive results. Obesity and BMI-related adipocytokines were not associated with immune dysregulation in patients with sepsis, and the relationship between resistin and outcomes was driven by inflammation rather than obesity itself [14].

Systematic reviews by Hajri et al. and Alipoor et al. have comprehensively reviewed leptin, adiponectin, resistin, visfatin, asymmetric dimethylarginine (ADMA), and ghrelin in critical illness [15,16]. Key findings include inconsistent associations between leptin and adiponectin and severity and mortality, and no consensus on their circulatory and functional changes [15,16]. As for resistin and visfatin, there was more consistent evidence of their elevation associated with inflammation, organ failure, and mortality [15,17]. Preclinical studies suggest a protective role for adiponectin, but clinical findings present a complex picture with inconsistent correlations [18]. The most recent comprehensive review by Joshi et al. focused exclusively on leptin, adiponectin, and resistin, confirming that these three classical adipokines dominate the literature on adipokine–immunity–infection interactions [7]. Critically, Alipoor et al. concluded that “further studies are required to clarify whether the reason of these changes is pathophysiological or compensatory” [16].

The last two decades have witnessed the identification of a new generation of adipokines, including chemerin, vaspin, and omentin-1. Each possesses distinct structural and functional characteristics, with experimental evidence from cell-based and animal models providing mechanistic insight and clinical cohort data offering biomarker validation. This review examines the biochemistry, experimental models, and clinical evidence for all three adipokines in the setting of sepsis and critical illness.

Chemerin functions in innate immune cell recruitment and antimicrobial defense. Vaspin acts as a serine protease inhibitor with anti-oxidative and anti-apoptotic properties. Omentin-1 provides endothelial protection and cardiomyocyte mitochondrial quality control [19,20,21]. All three converge on 5′ AMP-activated protein kinase (AMPK), nuclear factor kappa-light-chain-enhancer of activated B cells (NF-κB), phosphoinositide 3-kinase (PI3K/protein kinase B (Akt), and NOD-, LRR- and pyrin domain-containing protein 3 (NLRP3) inflammasome pathways, central nodes in sepsis pathophysiology, providing a coherent mechanistic framework [3,22]. Kukla et al. specifically investigated chemerin, vaspin, and omentin-1 as a coherent group of “anti-inflammatory adipokines” in COVID-19, establishing a conceptual and methodological precedent [19]. The Ebihara et al. profiling study highlighted that chemerin and vaspin remain insufficiently characterized within the sepsis cytokine network, while omentin was not even included in their panel, a specific gap this review addresses [17]. Recent prospective studies have generated high-quality clinical data on chemerin and omentin-1 in sepsis that have not been supported by the preclinical literature [21,23].

Hence, this narrative review aims to comprehensively accumulate the current evidence on chemerin, vaspin, and omentin-1 in sepsis and acute lung inflammation, spanning in vitro mechanistic studies, in vivo experimental models, and clinical investigations in critically ill patients. The review examines the molecular signaling pathways through which these adipokines exert their immunomodulatory and organ-protective effects, evaluates their potential as diagnostic and prognostic biomarkers, and identifies translational gaps and future research directions.

## 2. Adipose Tissue in Critical Illness

### 2.1. The Adipose–Immune–Metabolic Axis in Sepsis

Critical illness induces a profound hypermetabolic and hyperinflammatory state, fundamentally altering the endocrine function of adipose tissue from a passive energy reservoir into an active immunometabolic hub [10]. Sympathetic nervous system activation, elevated catecholamines, glucocorticoids, and inflammatory cytokines such as tumor necrosis factor (TNF)-α, interleukin (IL)-6, and IL-1β collectively reprogram adipose tissue secretory behavior [24]. In early sepsis, the adipose tissue becomes a site of immune cell infiltration, particularly by pro-inflammatory M1 macrophages, contributing to the systemic cytokine storm [10,25]. Simultaneously, insulin resistance emerges as a near-universal feature of sepsis, mediated in part through adipokine dysregulation [26,27].

A recent review proposes three core mechanisms.

(1) Metabolic defense priority, in which adipose tissue mobilizes to spare skeletal muscle protein to provide fatty acids as fuel for immune cells and vital organs during the hypermetabolic state. (2) Bidirectional immunometabolic crosstalk, in which immune cells regulate adipose tissue lipolysis via IL-1β or transforming growth factor-β (TGF-β), while adipokines reciprocally modulate immune cell function. This creates feedback loops where pro-inflammatory adipokines amplify inflammation while anti-inflammatory adipokines serve as counter-regulatory molecules. (3) Stage-specific adaptation, which means that the adipokine profile evolves dynamically. Pro-inflammatory adipokines dominate acutely, while anti-inflammatory adipokines rise during recovery. Morphologically, adipose tissue of critically ill patients shows increased numbers of newly differentiated smaller adipocytes and a macrophage phenotypic switch toward the M2-type, suggesting an adaptive response [10].

**Figure 1:**
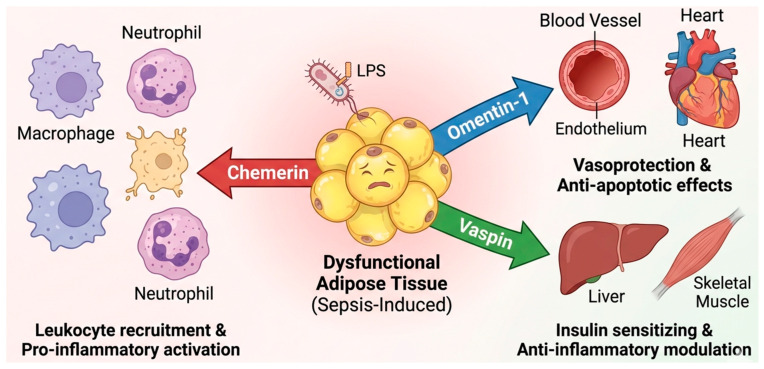
Adipose–organ crosstalk in sepsis. Schematic representation of the endocrine role of dysfunctional adipose tissue during the septic response. Under the stress of systemic infection (triggered by bacterial lipopolysaccharide—LPS), shrunken and dysfunctional adipocytes serve as an immunometabolic hub, releasing specific adipokines with divergent systemic effects. Pro-inflammatory axis (Red): Chemerin acts as a potent chemoattractant, signaling to macrophages and neutrophils to promote leukocyte recruitment and systemic inflammatory activation. Protective axis (Blue/Green): Conversely, omentin-1 (Blue) targets the vascular endothelium and myocardium to provide vasoprotection and anti-apoptotic effects, while vaspin (Green) acts on the liver and skeletal muscle to modulate insulin sensitivity and dampen the hyper-inflammatory cascade. This crosstalk highlights how adipose tissue can either exacerbate systemic injury or provide compensatory protective signals to vital organs.

Figure 1 illustrates the central concept of the review. How dysfunctional adipose tissue releases these three specific adipokines to act on the endothelium, heart, liver, and skeletal muscle during the septic response.

### 2.2. Key Experimental Models Used in Adipokine Research

In vitro models relevant to sepsis adipokine research include lipopolysaccharide (LPS)-stimulated macrophage cell lines (RAW264.7, U937, THP-1), human umbilical vein endothelial cells (HUVECs), human microvascular endothelial cells (HMVECs), human pulmonary microvascular endothelial cells (HPMECs), vascular smooth muscle cells (VSMCs), human aortic endothelial cells (HAECs), and 3T3-L1 adipocytes [28,29,30]. These allow mechanistic dissection of cytokine production, adhesion molecule expression, NF-κB activation, and barrier function. In vivo models include the cecal ligation and puncture (CLP) model, the gold standard for polymicrobial sepsis recapitulating the clinical trajectory, and intraperitoneal or intratracheal LPS administration for endotoxemia and acute lung injury/acute respiratory distress syndrome (ALI/ARDS) models, respectively [31]. The CLP model is particularly important for vaspin research, providing cardioprotective and mortality data, while LPS-ALI models have been pivotal for both vaspin and omentin-1 lung protection data [32,33,34].

The alterations render circulating adipokine levels highly dynamic during critical illness, varying with both disease severity and temporal kinetics throughout the illness trajectory [6]. Because adipokines integrate metabolic and immune signals, their measurement may provide multidimensional pathophysiological information beyond single-analyte biomarkers. The theoretical appeal of this is well recognized, and studying novel adipokines in experimental sepsis models has become an important strategy for understanding their mechanistic contribution to disease and evaluating their therapeutic potential.

## 3. Chemerin

### 3.1. Biochemistry and Molecular Biology

Chemerin is encoded by the retinoic acid receptor responder 2 (*RARRES2*) gene and is synthesized as an inactive 163-amino acid preprotein, prochemerin [35]. Following removal of a 20-amino acid N-terminal signal peptide, the mature form undergoes C-terminal proteolytic processing by inflammatory and coagulation serine proteases, including plasmin, neutrophil elastase, and cathepsins, generating a spectrum of isoforms with varying receptor affinities. The most biologically potent isoform, chemerin 21–157, serves as the primary endogenous ligand for the chemokine-like receptor 1 (CMKLR1), also known as chemerin receptor 23 (ChemR23) or chemerin receptor 1, a G protein-coupled receptor (GPCR) highly expressed on innate immune cells including plasmacytoid dendritic cells (pDCs), macrophages, and natural killer (NK) cells [36]. This activation of prochemerin by coagulation serine proteases is particularly relevant in sepsis, where simultaneous coagulation cascade activation may drive autocrine and paracrine chemerin signaling at sites of infection [37,38].

Chemerin is produced not only by adipocytes but also by hepatocytes, fibroblasts, and epithelial cells of the lung, kidney, adrenal gland, pancreas, and skin [21]. Its cationic regions can disrupt bacterial membranes, conferring direct antimicrobial properties. Additionally, chemerin regulates adipogenesis, glucose homeostasis, and insulin signaling in skeletal muscle and contributes to the development of insulin resistance, a common and clinically significant complication in sepsis [39].

### 3.2. Immunological Functions: Pro- and Anti-Inflammatory Duality

Chemerin signals through CMKLR1 (ChemR23), a Gαi-coupled GPCR highly expressed on macrophages, neutrophils, DCs, and NK cells [35]. Receptor signaling proceeds through Gαi1/i2/i3 and Gαo subtypes, followed by recruitment of β-arrestin 1 and 2. Downstream, MAPK/ERK1/2 and PI3K/Akt activation requires both Gαi/o and β-arrestin 2 [40,41].

ERK1/2 and PI3K/Akt converge on NF-κB activation, driving the expression of vascular cell adhesion molecule-1 (VCAM-1), intercellular adhesion molecule-1 (ICAM-1), E-selectin, and monocyte chemoattractant protein-1 (MCP-1) in endothelial cells, enhancing monocyte–endothelial adhesion [42]. IL-1β acts synergistically with chemerin to amplify NF-κB-mediated inflammation [42].

A critical nuance is that CMKLR1 signaling is context-dependent. Chemerin acts as a potent chemoattractant for pDCs and macrophages to sites of inflammation, facilitating early innate immune activation, promoting pro-inflammatory macrophage/DC recruitment. Conversely, CMKLR1 activation can also induce pro-resolving pathways. The nanopeptide chemerin9, derived from the C-terminus of active chemerin, has been shown to induce pro-resolving macrophage phenotype changes via Gi signaling, reducing inflammatory mediator production [35]. Resolvin E1 (RvE1), a specialized anti-inflammatory lipid mediator that inhibits leukocyte infiltration and pro-inflammatory gene expression, has also been proposed to signal through CMKLR1, further linking chemerin biology to inflammation resolution, a process critically dysregulated in sepsis [36,43]. Additionally, CMKLR1-deficient mice paradoxically show enhanced inflammation in some models, possibly because CMKLR1 also recruits tolerogenic pDCs [43]. This dual nature, pro-inflammatory via chemerin and pro-resolving via RvE1, establish CMKLR1 as a multifunctional receptor whose net effect depends on the ligand milieu [35,36,43].

Receptor internalization and desensitization are regulated by G protein-coupled receptor kinase 6 (GRK6) and β-arrestin 2. GRK6-deficient macrophages show increased migration toward chemerin and altered Akt/ERK signaling, suggesting that impaired receptor desensitization could amplify chemerin-driven inflammation in disease states [41]. Conversely, the nanopeptide chemerin9 and RvE1 signal through CMKLR1 to promote pro-resolving macrophage phenotypes and suppress inflammation [35,36].

Hence, chemerin occupies a paradoxical position in immunology; it can exert both pro- and anti-inflammatory effects depending on the physiological context, isoform generated, and receptor subtype engaged [36].

### 3.3. In Vitro Evidence

In vitro data on the immunological functions of chemerin have yielded complex and sometimes contradictory results, reflecting the isoform- and context-dependent nature of its biology.

In human endothelial cells, chemerin exhibits opposing effects depending on the experimental conditions. Chemerin has been shown to induce NF-κB activation in HUVECs via MAPK/ERK1/2 and PI3K/Akt pathways, driving expression of the adhesion molecules E-selectin, VCAM-1, and ICAM-1 [42]. It has also been shown to enhance monocyte–endothelial adhesion in functional assays, a critical early step in atherosclerosis and vascular inflammation [42]. Moreover, IL-1β acts synergistically with chemerin to amplify NF-κB-mediated inflammation, suggesting chemerin potentiates cytokine-driven endothelial activation [42]. Conversely, chemerin has been shown to inhibit TNF-α-induced VCAM-1 expression in HUVECs by activating the Akt/endothelial nitric oxide synthase (eNOS) pathway and increasing nitric oxide (NO) production [44]. Furthermore, the same study showed that chemerin rapidly phosphorylated Akt and eNOS, increasing intracellular cGMP. This, in turn, suppressed TNF-α-induced phosphorylation of NF-κB p65 and p38 MAPK, reducing VCAM-1 expression and monocyte adhesion. The protective effect was NO-dependent, since NOS inhibitors could reverse chemerin’s anti-inflammatory actions, while NO donors mimicked them. Similar anti-inflammatory effects were observed in rat isolated aorta ex vivo [44]. These opposing effects likely reflect timing and context. Acute chemerin exposure (minutes) activates Akt/eNOS/NO signaling with anti-inflammatory consequences, while prolonged exposure or co-stimulation with IL-1β activates MAPK/NF-κB pathways with pro-inflammatory outcomes. Hence, chemerin concentration, endothelial cell activation state, and the presence of other inflammatory mediators determine the net effect.

In HMVECs and VSMCs, chemerin promotes pro-inflammatory and proliferative responses by inducing NADPH oxidase (NOX)-dependent reactive oxygen species (ROS) production [45]. In this study, chemerin increased ROS production and phosphorylation of MAPK (ERK1/2, p38, JNK), effects blocked by NOX inhibitors and the ROS scavenger N-acetylcysteine. In VSMCs, chemerin stimulated proliferation via redox-sensitive MAPK signaling, decreased PI3K/Akt activation, and increased TUNEL-positive VSMCs, indicating pro-apoptotic effects. In HMVECs, chemerin reduced eNOS activity and NO production, impairing endothelial function. Moreover, chemerin increased the mRNA expression of pro-inflammatory mediators (IL-6, IL-8, MCP-1) and enhanced monocyte-to-endothelial cell attachment. Finally, adipocyte-conditioned medium from obese/diabetic mice, which have elevated chemerin, increased ROS generation in VSMCs, while medium from control mice had no effect. These effects were blocked by CCX 832, a ChemR23 antagonist, confirming CMKLR1 dependence [45].

In macrophage models, no direct effect has been demonstrated on LPS-induced cytokine production. Specifically, mouse peritoneal macrophages and human monocyte-derived macrophages have been shown to express functional ChemR23 [46]. However, using peritoneal macrophages generated from wild-type or *CMKLR1*^-/-^ knockout (KO) mice, the authors demonstrated that bioactive chemerin did not modulate cytokine responses despite functional ChemR23 expression, with identical null results in human blood monocyte-derived macrophages [46]. Hence, despite functional *CMKLR1* expression, chemerin did not seem to modulate LPS-induced cytokine production in macrophages. This contradicts earlier reports of direct anti-inflammatory effects of chemerin on macrophages and suggests that chemerin’s anti-inflammatory actions in vivo occur through indirect mechanisms, likely recruitment of tolerogenic pDCs or modulation of the tissue microenvironment rather than direct suppression of macrophage cytokine production [47].

While chemerin does not seem to affect macrophage cytokine production, it potently promotes macrophage adhesion to extracellular matrix and endothelium. Chemerin could stimulate adhesion of mouse peritoneal exudate cells to fibronectin and VCAM-1, via ChemR23 and Gαi signaling. Moreover, 89% of adhesion to fibronectin was mediated by increased avidity of integrin VLA-5 (α5β1), while 88% of adhesion to VCAM-1 was mediated by VLA-4 (α4β1). Chemerin did not increase integrin affinity but instead promoted integrin clustering, as visualized by confocal microscopy. Key signaling mediators included PI3K, Akt, and p38 MAPK. Pertussis toxin and *CMKLR1*^-/-^-KO macrophages confirmed Gαi-coupled receptor dependence. This rapid adhesion response, combined with chemotactic activity, suggests chemerin promotes both recruitment and retention of macrophages at inflammatory sites [48].

The atypical chemerin receptor, C-C motif chemokine receptor-like 2 (CCRL2), which binds chemerin but does not signal, is expressed on endothelial cells and regulates chemerin bioavailability [49]. CCRL2 and VCAM-1 were found co-upregulated in human and mouse vascular endothelial cells by pro-inflammatory stimuli (TNF-α, IL-1β, LPS) via NF-κB and JAK/STAT signaling. CCRL2 was constitutively expressed at high levels by pulmonary endothelial cells and at lower levels by liver endothelium. Liver, but not pulmonary, endothelial cells further upregulated CCRL2 in response to systemic LPS. It was demonstrated that CCRL2 bound chemerin and presented it to CMKLR1 on nearby leukocytes, enhancing local chemerin bioactivity. Moreover, plasma chemerin levels were elevated in *CCRL2*-KO mice and increased further after LPS treatment, confirming that CCRL2 regulates circulating chemerin levels. Chemerin binding to endothelial CCRL2 triggered robust adhesion of CMKLR1+ lymphoid cells (NK cells) through an α4β1 integrin/VCAM-1-dependent mechanism. Lastly, in LPS-induced acute lung inflammation, CMKLR1+ NK cell recruitment to airways was significantly impaired in *CCRL2*-KO mice, demonstrating that endothelial CCRL2 is required for efficient chemerin-mediated leukocyte recruitment in vivo [49].

In inflammatory macrophages, CMKLR1 signaling and function were tightly regulated by GRK6 and β-arrestin 2, which modulate receptor desensitization and internalization. Chemerin stimulation led to GRK6-mediated phosphorylation of CMKLR1 intracellular domains, recruitment of β-arrestin 2, and signaling termination. β-arrestin recruitment to CMKLR1 was enhanced by co-expression of GRK6. CMKLR1 internalization following chemerin stimulation was decreased in GRK6- and β-arrestin 2-deficient macrophages. These deficient macrophages displayed increased migration toward chemerin and altered Akt/ERK signaling, suggesting impaired receptor desensitization amplifying chemerin-driven responses. This regulatory mechanism may be therapeutically relevant, as defective GRK6/β-arrestin 2 function could lead to exaggerated chemerin-mediated inflammation in rheumatic diseases [41].

Finally, in peritoneal macrophages, *CMKLR1* expression was demonstrated to be dynamically regulated by inflammatory stimuli. Pro-inflammatory cytokines and Toll-like receptor (TLR) ligands suppressed macrophage CMKLR1 expression, while TGF-β upregulated the receptor. This stimulus-specific regulation might suggest that *CMKLR1* expression is downregulated during acute but upregulated during resolution [50].

In Table 1, the findings from the in vitro septic/inflammatory models studying chemerin are listed.

The in vitro data reveal that chemerin’s role in sepsis cannot be reduced to simply pro-inflammatory or anti-inflammatory. Instead, chemerin acts as a context-dependent immunomodulator whose effects depend on the cell type: pro-inflammatory in endothelial cells and VSMCs (via NOX/ROS/MAPK), anti-inflammatory in whole lung tissue (via pDC recruitment), neutral in macrophage cytokine production. Timing is important, as acute exposure activates protective Akt/eNOS/NO pathways, while chronic exposure or co-stimulation with IL-1β/TNF-α activates MAPK/NF-κB inflammation. Receptor regulation is another important component. CMKLR1 is downregulated by TLR ligands during acute inflammation but upregulated by TGF-β during resolution, suggesting chemerin’s role shifts across sepsis phases. Receptor type comprises another aspect of chemerin’s role. The signaling receptor CMKLR1 mediates direct cellular effects, while the non-signaling CCRL2 on endothelium concentrates chemerin to enhance local bioactivity. This complexity may explain why circulating chemerin is elevated in sepsis and predicts mortality. Yet, ChemR23-KO mice show worse outcomes in LPS models, so chemerin likely exerts both harmful (endothelial activation, ROS generation) and beneficial (tolerogenic DC recruitment) effects simultaneously, with the net outcome determined by disease stage and tissue context.

### 3.4. In Vivo Experimental Evidence

Animal models have provided important insights into the role of the chemerin/CMKLR1 axis in inflammatory lung disease and infection, conditions that are highly relevant to the pathophysiology of sepsis-associated organ injury.

In contrast to the mixed in vitro findings, chemerin exhibits potent anti-inflammatory effects in mouse LPS-induced lung inflammation. In a murine model of LPS-induced acute lung injury, the administration of exogenous chemerin acted as a protective agent by significantly reducing neutrophil infiltration and the release of inflammatory cytokines. This anti-inflammatory activity is strictly dependent on the ChemR23 (CMKLR1) receptor, as *CMKLR1*-KO mice failed to respond to chemerin treatment and instead demonstrated increased neutrophil accumulation following an LPS challenge. The primary mechanism for this protection likely involved the recruitment of tolerogenic pDCs, which express high levels of the ChemR23 receptor. While expression is highest in immature pDCs, the receptor is also present at lower levels on myeloid DCs, macrophages, and NK cells. This functional study indicated that chemerin promotes essential immune responses such as calcium mobilization and chemotaxis in these cells, both of which are entirely abrogated in *CMKLR1*-deficient models [47].

In a model of acute viral pneumonia using the pneumonia virus of mice (PVM), *CMKLR1*-KO mice exhibited higher mortality and morbidity, altered lung function, delayed viral clearance, reduced pDC recruitment, and diminished type I interferon production compared to wild-type controls, establishing the chemerin/CMKLR1 axis as an important mediator of anti-viral innate immunity [51]. In LPS-induced lung inflammation, *CMKLR1*-KO mice showed exacerbated pulmonary inflammatory responses [51].

Recombinant chemerin at picomolar concentrations has been reported to exert anti-inflammatory effects on zymosan-induced murine peritonitis in a proteolysis-dependent manner, by reducing pro-inflammatory mediator expression [52]. More specifically, chemerin15 (C15) was shown to inhibit macrophage activation to a similar extent as proteolyzed chemerin. Intraperitoneal administration of C15 to mice before zymosan challenge conferred significant protection against zymosan-induced peritonitis, suppressing neutrophil and monocyte recruitment with a concomitant reduction in pro-inflammatory mediator expression. Importantly, C15 was unable to ameliorate zymosan-induced peritonitis in *CMKLR1*-KO mice, demonstrating that C15’s anti-inflammatory effects are entirely ChemR23-dependent [52]. The same group demonstrated that during peritoneal inflammation, C15 administration enhanced microbial particle clearance and apoptotic neutrophil ingestion (efferocytosis) by macrophages in wild-type but not *CMKLR1*-KO mice, profoundly reducing levels of apoptotic and necrotic cells at the inflammatory site [53].

*CCRL2*-KO mice displayed exaggerated local and systemic inflammatory responses in both zymosan- and thioglycollate-induced peritonitis, characterized by increased myeloid cell recruitment. This amplified inflammation was associated with increased circulating and local chemerin levels. Antibody neutralization of chemerin in *CCRL2*-KO mice abrogated the amplified inflammatory responses, confirming that the phenotype is chemerin-dependent [54].

In another study, *CCRL2*-KO mice exhibited impaired NK cell recruitment in LPS-induced lung inflammation. Plasma chemerin levels were elevated in *CCRL2*-KO mice and further enhanced after systemic LPS, confirming CCRL2’s role in regulating circulating chemerin levels. This demonstrates that endothelial CCRL2 is required for efficient local concentration of chemerin at inflammatory sites to recruit CMKLR1^+^ immune cells [49].

Moreover, cathepsin K- and L-truncated chemerin displayed direct antibacterial activity against Enterobacteriaceae in addition to triggering robust migration of human pDCs ex vivo [55]. Another study using single-cell (sc)RNA sequencing in an ALI model identified that reverse-migrated neutrophils (those migrating away from the inflammatory site back into the vasculature) exhibited increased *CCRL2* expression. Circulating chemerin concentrations increased in the late stage of inflammation, and neutralizing chemerin decreased the reverse-migrated neutrophil ratio in blood, suggesting chemerin/CCRL2 interaction promotes neutrophil reverse migration, a mechanism potentially involved in dissemination of inflammation [56].

RvE1 serves as a ChemR23 ligand and acts as an endogenous pro-resolving lipid mediator. Administration of RvE1 6 h post-LPS in rats improved survival, increased alveolar fluid clearance, reduced lung wet–dry weight ratio, and mitigated lung injury scores [57]. In bacterial pneumonia models, RvE1 selectively decreased lung neutrophil accumulation, enhanced *E. coli* clearance, and markedly improved survival. Mechanistically, RvE1 seemed to limit collateral lung damage by independently downregulating pro-inflammatory cytokines such as IL-1β, IL-6, and high-mobility group box 1 (HMGB1) without impairing pathogen killing [58].

In a pulmonary inflammation model, RvE1 promoted phagocytosis-induced neutrophil apoptosis via the leukotriene B4 receptor 1 (BLT1), enhancing NADPH oxidase-derived ROS and caspase-8/3 activation, while attenuating anti-apoptosis signals from myeloperoxidase (MPO) and serum amyloid A (SAA) [59].

These discordant findings likely reflect differences in experimental design, genetic background, and the specific chemerin isoforms tested, and underscore that the direct anti-inflammatory effect of chemerin on macrophages cannot be generalized across all in vitro settings. Notwithstanding these complexities, chemerin has been consistently shown to drive chemotaxis of pDCs, NK cells, and immature DCs through CMKLR1 in transwell migration assays, establishing its role as a potent chemoattractant in inflammatory conditions [36]. Structural studies using cryo-electron microscopy (cryo-EM) have elucidated the molecular basis of CMKLR1 signaling by chemerin9, revealing agonist-induced conformational changes in the receptor that activate Gi signaling pathways, and providing a structural framework for the development of small-molecule CMKLR1 agonists that could promote resolution of inflammation [35].

Table 2 lists the chemerin studies in in vivo models of sepsis and organ injury.

These in vivo findings collectively support a context-dependent role for chemerin in infection and inflammation: pro-inflammatory and chemoattractive in early innate immune mobilization, but capable of promoting resolution when appropriate receptor signaling is engaged.

### 3.5. Clinical Evidence in Sepsis and Critical Illness

The most comprehensive prospective clinical data on chemerin in sepsis come from Karampela et al., who measured serum chemerin in 102 critically ill patients with sepsis within 48 h of onset and again one week later, compared to 102 age- and sex-matched healthy controls [21]. Serum chemerin was markedly elevated at sepsis onset and, while it declined significantly over the first week, it remained above control levels throughout follow-up. Levels were substantially higher in patients with septic shock than in those with sepsis alone, and in non-survivors compared to survivors at both timepoints. Crucially, Cox proportional hazards regression analysis revealed that elevated chemerin at admission was an independent predictor of 28-day mortality, yielding a Hazard Ratio (HR) of 3.58 (95% CI: 1.48–8.65, *p* = 0.005). When evaluated dynamically at one-week post-onset, the prognostic power of sustained chemerin elevation strengthened dramatically, escalating to an HR of 10.01 (95% CI: 4.32–23.20, *p* < 0.001), underlining its utility in tracking ongoing, unresolved systemic inflammation. The diagnostic performance for severity discrimination (AUC 0.78) was comparable to CRP. Chemerin correlated significantly with the acute physiology and chronic health evaluation (APACHE) II and the sequential organ function assessment (SOFA) scores, white blood cell (WBC) count, lactate, CRP, and procalcitonin [21].

A critical nuance was established by Horn et al. in peritoneal sepsis. In this study, chemerin correlated with intraoperative glucose, positioning it as a metabolic biomarker [60]. Crucially, the prognostic relationship was context-dependent; among patients with stress hyperglycemia (SHG), non-survivors had paradoxically lower chemerin, while non-survivors without SHG trended toward higher chemerin. Despite elevated circulating levels, visceral adipose *RARRES2* mRNA was decreased in sepsis, suggesting extra-adipose sources or altered clearance [60]. This paradoxical finding suggests that chemerin may serve different functions depending on metabolic context. In SHG, higher chemerin may reflect a compensatory insulin-sensitizing response that is protective, while in non-SHG patients, elevated chemerin may reflect greater inflammatory burden.

Amend et al. further showed that Gram-positive infection was associated with significantly higher plasma chemerin than Gram-negative infection or COVID-19, raising the possibility that chemerin could serve as an early biomarker to distinguish infecting organism class, a distinction with direct therapeutic implications for empiric antibiotic selection [61]. Importantly, patients with liver cirrhosis had markedly lower chemerin, highlighting the need to adjust for hepatic function when interpreting circulating levels in heterogeneous ICU populations [61].

Multiple studies have evaluated chemerin in COVID-19, providing the closest clinical analog to sepsis-associated ARDS. In 88 COVID-19 patients (40 ICU), plasma chemerin was significantly higher in ICU patients than healthy controls at all time points and higher in non-survivors than survivors. Moreover, the multivariate analysis showed that chemerin at day 14 was an independent risk factor for death. Immunohistochemistry of autopsied COVID-19 lungs revealed strong ChemR23 expression on smooth muscle cells and chemerin expression on myofibroblasts in advanced ARDS, suggesting active chemerin/ChemR23 signaling in the fibroproliferative phase [62]. A separate study confirmed sustained chemerin elevation in hospitalized COVID-19 patients with a trend toward further increase over 7 days [63]. However, one study reported decreased chemerin in COVID-19 patients, highlighting inconsistency across cohorts, likely reflecting differences in disease severity, timing of sampling, and assay methodology [19]. Thus, in SARS-CoV-2 infection, anti-inflammatory adipokines including chemerin are altered relative to controls, though direction seems to vary by disease severity.

A recent study provided novel insights into the resolution pathway in critically ill COVID-19 patients. Among a panel of cytokines and resolvins, RvE1 was the single best discriminator of COVID-19 severity, outperforming all cytokines, including IL-6. RvE1 was paradoxically elevated in the most severe patients, mechanically ventilated patients, and non-survivors, suggesting failed resolution rather than insufficient resolvin production. *CMKLR1* mRNA exhibited an opposite profile; higher expression correlated with lower inflammation, better respiratory function, and shorter hospital stay. This RvE1–ChemR23 axis dysregulation suggests that in severe ARDS, the resolution machinery is activated but functionally impaired, possibly due to receptor downregulation or desensitization [64].

Ebihara et al. performed a comprehensive adipocytokine profiling study on 37 septic patients with serial measurements over 15 days. Hierarchical clustering analysis revealed that chemerin does not cluster with the core inflammatory network (IL-6, IL-8, MCP-1, IL-10) dominated by resistin, suggesting it reflects a parallel immunometabolic pathway offering complementary prognostic information [17].

## 4. Vaspin (SERPINA12)

### 4.1. Biochemistry and Molecular Biology

Vaspin, visceral adipose tissue-derived serine protease inhibitor, is designated *SERPINA12*, encoding a 47 kDa serine protease inhibitor first identified in 2005 in visceral adipose tissue of a rat diabetes model [65]. In humans, vaspin is predominantly expressed in visceral adipose tissue but also in the heart, kidney, brain, gastrointestinal tract, pancreas, and skin [65]. As a serpin, vaspin inhibits serine protease activity through a suicide inhibitor mechanism, forming a covalent complex with its target protease. Kallikrein 7 (KLK7) was identified as its primary confirmed target via classical serpin inhibition, with covalent vaspin–KLK7 complexes detectable in human plasma [66]. A landmark structural study by Möhlis et al. revealed that vaspin binds DNA with high affinity, accelerating KLK7 inhibition approximately 5-fold and potentially contributing to intracellular nuclear effects following low-density lipoprotein receptor-related protein 1 (LRP1)-mediated internalization, significantly expanding understanding of vaspin’s biological reach [67]. Additional cell surface interactions with glucose-regulated protein 78 kDa (GRP78), also known as binding immunoglobulin protein (BiP), encoded by the *HSPA5* gene, have been described, mediating PI3K/Akt pathway activation and contributing to vaspin’s anti-apoptotic and insulin-sensitizing functions [65].

### 4.2. Immunological and Metabolic Functions

Vaspin is principally an anti-inflammatory adipokine. It has been shown to suppress expression of pro-inflammatory cytokines including TNF-α, IL-6, and IL-1β in macrophages and vascular cells, and inhibit the NF-κB pathway via 5′ adenosine monophosphate-activated protein kinase (AMPK) activation [65]. It has also been shown to inhibit apoptosis induced by free fatty acids in vascular endothelial cells, and in adipose tissue it reciprocally modulated circulating levels of other adipokines including adiponectin and resistin [68]. It seems, therefore, that vaspin promotes insulin secretion from pancreatic β-cells and acts as an insulin sensitizer, establishing it as a node in the metabolic-immune network particularly relevant to the stress hyperglycemia of critical illness.

### 4.3. In Vitro Evidence

Vaspin in in vitro septic models demonstrates predominantly anti-inflammatory and cytoprotective effects across multiple cell types, though with notable cell-type-dependent discrepancies that parallel the contradictions seen with chemerin. The evidence spans endothelial cells, cardiomyocytes, VSMCs, adipocytes, and renal tubular epithelial cells.

In HAECs, vaspin significantly increased AMPK phosphorylation and, through this mechanism, reduced TNF-α-induced NF-κB activation and downstream expression of adhesion molecules ICAM-1, VCAM-1, E-selectin, and MCP-1. These effects were abolished by AMPKα1-specific siRNA knockdown, confirming AMPK as the operative upstream mediator [28]. Consistent findings were obtained in human endothelial EA.hy926 cells, where vaspin pretreatment inhibited TNF-α- and IL-1-stimulated NF-κB transcriptional activity and reduced downstream cytokine production in a dose-dependent manner [69]. In direct contrast, vaspin had no effect on basal HUVEC morphology or TNF-α-induced morphological damage. More specifically, vaspin did not inhibit TNF-α-induced activation of JNK, p38, or NF-κB, and did not decrease TNF-α-induced expression of VCAM-1, ICAM-1, E-selectin, COX-2, MCP-1, tissue factor, or plasminogen activator inhibitor-1 (PAI-1) [70].

The divergent results likely reflect differences in endothelial cell origin and phenotype (aortic vs. umbilical vein vs. hybrid EA.hy926), vaspin concentration ranges (up to 320 ng/mL in positive studies vs. 100 ng/mL in the negative study), and receptor expression profiles. HAECs may express higher levels of vaspin-responsive receptors (GRP78, LRP1) than HUVECs. This cell-type specificity is clinically relevant because sepsis-induced endothelial dysfunction varies across vascular beds; pulmonary, hepatic, and renal microvascular endothelium may respond differently to vaspin than large-vessel endothelium.

Of particular relevance to sepsis-associated acute lung injury (ALI); in vitro experiments in HPMECs demonstrated that recombinant vaspin pretreatment could reverse LPS-induced upregulation of TNF-α, IL-6, VCAM-1, and E-selectin mRNA, and reduce phosphorylation and nuclear translocation of the NF-κB Rel subunit at 2 h post-LPS insult [33].

In rat mesenteric artery VSMCs, vaspin exerted potent anti-inflammatory effects by targeting a specific ROS-dependent signaling axis. While it did not alter VSMC morphology, it effectively prevented monocyte adhesion by suppressing TNF-α-induced ICAM-1 expression. The core mechanism involved the inhibition of ROS generation, which subsequently halted the downstream phosphorylation of NF-κB and protein kinase C theta (PKCθ). The antioxidant N-acetyl-L-cysteine (NAC) blocked TNF-α-induced NF-κB, PKCθ, and ICAM-1 activation, confirming that ROS acts upstream of both NF-κB and PKCθ. This discovery is significant because it establishes a novel pathway in vascular smooth muscle cells that is distinct from the AMPK-dependent mechanisms previously observed in endothelial cells [71].

Vaspin has been extensively studied in H9C2 rat cardiomyoblasts under various injury conditions relevant to sepsis-induced cardiomyopathy. Administration of TNF-α inhibited autophagy and promoted apoptosis in H9C2 cells. Vaspin pretreatment significantly mitigated apoptosis by augmenting autophagy. The mechanism involved inhibition of the PI3K/Akt/mammalian target of rapamycin (mTOR) pathway. Moreover, the Akt agonist, insulin-like growth factor-1 (IGF-1), reversed vaspin’s pro-autophagic effects, confirming that vaspin promotes autophagy by suppressing Akt/mTOR signaling [72]. In hypoxia/reoxygenation (H/R) injury, recombinant vaspin suppressed H/R-induced apoptosis in cardiomyocytes through AMPK-mTOR-dependent activation of autophagic flux. Blockage of autophagic flux with chloroquine mitigated vaspin’s protective effects, confirming that autophagy is the essential mediator [73]. In a separate study, vaspin ameliorated H/R injury in H9C2 cells in a dose-dependent manner. Vaspin reduced IL-1β, IL-18, and TNF-α, downregulated TLR4 expression, and reduced NF-κB phosphorylation in H/R-induced H9C2 cells, identifying the TLR4/NF-κB axis as a direct target [74]. In H9C2 cells exposed to high glucose (modeling diabetic cardiomyopathy), vaspin attenuated mitochondrial ROS generation and mitochondrial membrane depolarization. It inhibited NLRP3 inflammasome activation, reducing caspase-1 cleavage and IL-1β/TNF-α maturation. This NLRP3 suppression was autophagy-dependent, since the autophagy inhibitor 3-methyladenine (3-MA) abolished vaspin’s inhibitory effect on NLRP3 activation [75].

In 3T3-L1 murine adipocytes with stable vaspin expression, IL-1β-induced expression and secretion of IL-6, MCP-1, and TNF-α were significantly blunted. Exogenous vaspin treatment reduced cytokine-induced activation of the IKKα/β/IκB/NF-κB signaling cascade. Endogenous vaspin expression also enhanced insulin signaling by increasing insulin-stimulated Akt phosphorylation, linking anti-inflammatory and insulin-sensitizing effects. Adipogenic marker genes and lipid accumulation were similar to controls, indicating that vaspin selectively modulates inflammation without disrupting adipocyte biology [75]. Vaspin’s effects in adipocytes are relevant to sepsis because adipose tissue is a major source of inflammatory mediators during critical illness.

In HK-2 cells subjected to H/R, vaspin reduced expression of endoplasmic reticulum (ER) stress markers and the pro-inflammatory alarmin HMGB1, a key damage-associated molecular pattern (DAMP) in sepsis. Through HMGB1 inhibition, vaspin activated the Nrf2/antioxidant response element (ARE)/heme oxygenase-1 (HO-1) signaling pathway, an antioxidant defense mechanism, while simultaneously inhibiting the NF-κB signaling pathway. In the corresponding in vivo model, vaspin-treated mice showed reduced renal tubular edema, decreased urinary injury markers, reduced serum inflammatory factors, and lower renal oxidative stress [76]. This model is relevant to sepsis-associated kidney injury (AKI), as it models renal I/R injury.

A critical unresolved question is which receptor mediates vaspin’s cellular effects, with implications for understanding its mechanism in sepsis. Vaspin was identified as a ligand for cell-surface GRP78/MTJ-1 complex in hepatocytes. It increased phosphorylation of Akt and AMPK in a dose-dependent manner, and anti-GRP78 antibodies completely abrogated vaspin-induced pAkt and pAMPK upregulation [77]. In HAECs, vaspin binds the GRP78/voltage-dependent anion channel (VDAC) complex on the plasma membrane with high affinity. This binding is enhanced by ER stress, which recruits GRP78 from the ER to the cell surface [78].

More recent work has challenged the GRP78 model, demonstrating that vaspin internalization in adipocytes occurs by clathrin-mediated endocytosis dependent on low-density lipoprotein receptor-related protein 1 (LRP1), not GRP78. Vaspin has nanomolar affinity for LRP1 clusters II–IV, and binding to cell-surface heparan sulfates is required for efficient LRP1-mediated internalization. However, only native (not cleaved) vaspin is efficiently endocytosed, and internalized vaspin is ultimately targeted for lysosomal degradation. Vaspin internalization is increased in mature adipocytes after insulin-stimulated translocation of LRP1, suggesting metabolic state-dependent receptor availability [79].

The current model suggests that GRP78 mediates vaspin’s signaling effects (Akt/AMPK activation), while LRP1 mediates vaspin’s endocytosis and clearance. Both receptors may operate simultaneously in different cellular compartments [77,79].

Unlike chemerin, which shows bidirectional effects (pro- and anti-inflammatory depending on context), vaspin is almost uniformly anti-inflammatory and cytoprotective across in vitro models. The one exception, the negative HUVEC study, likely reflects cell-type-specific receptor expression rather than a true pro-inflammatory action [70]. Additionally, vaspin’s mechanisms are more diverse than chemerin’s. Vaspin operates through at least five distinct pathways (AMPK/NF-κB, Akt/glycogen synthase kinase-3 beta (GSK-3β), ROS/NF-κB/PKCθ, PI3K/Akt/mTOR/autophagy, and HMGB1/nuclear factor erythroid-2-related factor 2 (Nrf2), whereas chemerin primarily signals through CMKLR1/MAPK/PI3K-Akt. This mechanistic diversity may explain why vaspin shows protective effects across multiple organ systems (heart, lung, kidney, vasculature) in sepsis-relevant models. The major limitation is that no study has directly tested vaspin in LPS-stimulated macrophages or immune cells, a critical gap given that macrophage activation is central to sepsis pathophysiology. Whether vaspin modulates macrophage cytokine production, polarization, or phagocytic function remains unknown.

Table 3 lists all studies on vaspin in in vitro septic/inflammatory models.

### 4.4. In Vivo Experimental Evidence

The most mechanistically informative in vivo evidence for vaspin in sepsis comes from the study by Yin et al. (2022) using murine CLP and LPS models [34]. Septic mice exhibited markedly increased vaspin expression in both cardiac tissue and serum compared to sham-treated animals. Pre-treatment with recombinant vaspin prior to CLP significantly reduced 7-day mortality, attenuated cardiac injury biomarker elevation, improved left ventricular function, and reduced infiltration of CD45+ and CD68+ inflammatory cells into cardiac tissue. Cardiomyocyte apoptosis was also significantly reduced. Crucially, in *KLK7*-KO mice, the protective effects of vaspin on cardiac function, inflammatory cell infiltration, and apoptosis were completely abolished, confirming that KLK7 inhibition is the operative downstream mechanism for vaspin-mediated cardioprotection in sepsis [34].

Complementing these cardiac findings, vaspin has demonstrated protection against LPS-induced ALI in vivo. In a murine model of LPS-induced ALI/ARDS, systemic vaspin administration significantly attenuated pulmonary inflammatory responses, reducing lung wet/dry weight ratios, inflammatory cell infiltration in bronchoalveolar lavage fluid (BALF), and histological lung injury scores [33]. Mechanistically, pulmonary vaspin exerted its protective effects via the Akt/GSK-3β signaling pathway, reducing endothelial permeability, inflammatory cytokine levels, and apoptosis in lung tissue [33].

In a murine model of myocardial ischemia/reperfusion (I/R) injury, which shares key mechanistic features with septic cardiomyopathy, including oxidative stress and NF-κB-driven inflammation, systemic delivery of adeno-associated virus-vaspin (AAV-vaspin) reduced myocardial infarct size and apoptosis, and improved cardiac function after reperfusion [73]. In a rat I/R model, vaspin exerted cardioprotection through TLR4/NF-κB pathway inhibition [74]. In a rat model of diabetic cardiomyopathy induced by streptozotocin (STZ), vaspin treatment improved cardiac function, reduced cardiomyocyte apoptosis, and improved myocardial tissue and mitochondrial morphology. Moreover, it augmented autophagy and inhibited NLRP3 inflammasome activation. The autophagy inhibitor 3-MA abolished vaspin’s inhibitory effect on NLRP3, establishing the pathway [75]. In another study, vaspin reversed cardiac dysfunction by promoting autophagy through inhibition of the PI3K/Akt/mTOR pathway [72]. In C57BL/6 mice subjected to renal I/R injury, subcutaneous injection of recombinant mouse vaspin significantly improved renal tubular epithelial cell edema, decreased urinary injury markers, and reduced serum inflammatory factors and renal oxidative stress levels [76]. Vaspin transgenic mice showed ameliorated intimal proliferation in cuff-injured femoral arteries. Adenoviral vaspin ameliorated intimal proliferation of balloon-injured carotid arteries in diabetic Wistar rats, with reduced C-C motif chemokine ligand 2 (*CCL2*), platelet-derived growth factor subunit B (*PDGFB*), and platelet-derived growth factor receptor beta (*PDGFRB*) gene expression [78]. Finally, Ji et al. studied the effects of vaspin administration in three distinct in vivo heart failure (HF) models in rats (ischemic, pressure overload, neurohormonal). Across all three models, vaspin treatment alleviated cardiac fibrosis, demonstrating that its cardioprotective effects are not model-specific but rather a generalizable phenomenon across different heart failure etiologies [80].

A critical observation from comparing these studies is that vaspin’s relationship with PI3K/Akt signaling is context-dependent. This apparent paradox is explained by Packer’s comprehensive review, which notes that vaspin acts by “activating AMPK and suppressing PI3K-Akt-mTOR signaling” to enhance autophagy and reduce organellar stress, while inhibiting maladaptive cardiac hypertrophy and pro-inflammatory signaling [81].

Table 4 presents the studies on vaspin in vivo models of sepsis and organ injury.

### 4.5. Clinical Evidence in Sepsis and Critical Illness

Clinical evidence on vaspin in sepsis remains exploratory relative to the other two adipokines reviewed here. Vaspin has the least clinical data of the three adipokines in sepsis/critical care settings. No large prospective clinical study has specifically measured vaspin in septic ICU patients.

The foundational clinical study by Motal and colleagues measured plasma vaspin in 57 ICU patients meeting ACCP/SCCM sepsis criteria [82], compared to 48 critically ill control patients admitted for trauma or major surgery, matched for age, sex, weight, and diabetes status [83]. Vaspin plasma concentrations were significantly elevated in septic patients compared to controls, consistent with an acute-phase response. While the study was exploratory, the finding of elevated vaspin in human sepsis parallels the experimental evidence and supports the translational relevance of murine model findings [83].

The Kukla et al. study is the only clinical study that simultaneously measured all three adipokines (chemerin, vaspin, omentin) in critically ill patients. In 70 COVID-19 patients vs. 20 healthy controls, vaspin concentrations did not differ between groups. Vaspin showed no correlation with COVID-19 severity (pneumonia, dyspnea, ICU admission), inflammatory markers, or liver dysfunction. This null finding contrasts sharply with the significant changes observed for chemerin and omentin in the same cohort [19].

While not directly related to sepsis, several clinical observations are relevant. The broader clinical literature on vaspin in cardiovascular and metabolic disease reviewed by Dąbrowski et al. identifies its atheroprotective, anti-apoptotic, and insulin-sensitizing properties as highly pertinent to the multi-organ dysfunction syndrome of sepsis [65]. The influence of renal function on circulating vaspin clearance must also be considered, as acute kidney injury (AKI), common in sepsis, may independently raise vaspin levels, potentially confounding their interpretation as a disease-specific biomarker. Packer further contextualized vaspin within an adipokine hypothesis of cardiac dysfunction, linking its biology to the inflammation-driven cardiometabolic failure seen in both heart failure with preserved ejection fraction (HFpEF) and septic cardiomyopathy [81].

The absence of dedicated clinical sepsis studies for vaspin represents a critical translational gap. The robust preclinical evidence (CLP sepsis model showing reduced mortality with vaspin via KLK7 inhibition and LPS-ALI protection via Akt/GSK-3β) has not been validated in human sepsis cohorts.

## 5. Omentin-1 (Intelectin-1)

### 5.1. Biochemistry and Molecular Biology

Omentin-1, also known as intelectin-1, is a 313-amino acid glycoprotein predominantly secreted by the stromal vascular fraction of visceral adipose tissue, with additional expression in epicardial fat depots, intestinal goblet cells, and bronchial epithelium [84]. Two isoforms exist, omentin-1 and omentin-2, with omentin-1 being the dominant circulating form [85]. As a member of the intelectin family, omentin-1 is a calcium-dependent lectin capable of binding galactofuranose-containing carbohydrates found on the surface of bacterial and fungal pathogens, suggesting a role in innate immune pattern recognition that is directly relevant in the setting of sepsis [23]. Omentin-1 circulates as a homotrimeric complex and signals through multiple pathways, including AMPK activation, Akt phosphorylation, inhibition of NF-κB, and modulation of ERK, JNK, and p38 MAPK pathways [32]. Circulating levels are negatively regulated by insulin, glucose, leptin, and inflammatory cytokines, and positively by fibroblast growth factor 21 (FGF-21) and dexamethasone [84].

### 5.2. Immunological and Metabolic Functions

Omentin-1 has been shown to suppress endothelial adhesion molecule expression, including VCAM-1 and ICAM-1, via ERK/NF-κB pathway inhibition, thereby reducing leukocyte recruitment to the endothelium, a process central to the microvascular dysfunction of sepsis [86]. It has also been shown to promote M2 (anti-inflammatory) macrophage polarization, shifting the innate immune phenotype away from the M1-dominant, tissue-damaging response that characterizes uncontrolled sepsis [87]. In adipose tissue, omentin-1 was shown to suppress the thioredoxin-interacting protein (TXNIP)/NLRP3 inflammasome axis, and systemically reduce circulating pro-inflammatory cytokines, including TNF-α and IL-6 [32,88]. Additional protective mechanisms include stimulation of eNOS-dependent NO production via Akt/eNOS activation, inhibition of VSMC proliferation, activation of AMPK/peroxisome proliferator-activated receptor delta (PPARδ) signaling to reverse endothelial endoplasmic reticulum (ER) stress and ROS, and suppression of activin type II receptor and Wnt5a/Ca^2+^ signaling to improve mitochondrial biogenesis in cardiomyocytes [32,89,90]. Metabolically, omentin-1 has been shown to enhance insulin-stimulated glucose uptake in adipocytes independently of the PI3K pathway, providing a mechanism relevant to sepsis-associated stress hyperglycemia [91].

### 5.3. In Vitro Evidence

Omentin-1 in in vitro septic models demonstrates uniformly anti-inflammatory and cytoprotective effects across all cell types tested, a notable contrast to chemerin’s bidirectional actions and even vaspin’s occasional null results in HUVECs. Omentin-1 is the only one of the three adipokines with direct, confirmed anti-inflammatory effects on macrophages, filling a critical gap in the vaspin literature.

Multiple in vitro studies have characterized omentin-1 as a potent inhibitor of LPS-driven inflammatory signaling. In macrophage cell lines, omentin-1 has been shown to inhibit LPS-induced activation and phagocytic activities by suppressing TLR4/MyD88/NF-κB signaling, reducing downstream production of TNF-α, IL-1β, and IL-6 [30].

In RAW 264.7 macrophages stimulated with LPS, omentin-1 pretreatment inhibited inflammation via the TXNIP/NLRP3 signaling pathway. Omentin-1 reduced TXNIP, which normally activates the NLRP3 inflammasome by dissociating from thioredoxin under oxidative stress. In turn, NLRP3 inflammasome suppression reduced caspase-1 activation and downstream IL-1β and IL-18 maturation, key pyroptosis-associated cytokines in sepsis. The NLRP3 inhibitor MCC950 replicated omentin-1’s anti-inflammatory effects in the corresponding in vivo model, confirming NLRP3 as the essential downstream target [92].

In rheumatoid arthritis synovial fibroblasts, omentin-1 stimulation augmented IL-4 synthesis, which subsequently promoted M2 macrophage polarization in co-culture systems. The signaling cascade was confirmed by pharmacological inhibitors and siRNA knockdown. All pathway components partially reversed omentin-1-induced IL-4 production. In vivo, intra-articular omentin-1 injection blocked collagen-induced arthritis by upregulating IL-4 and M2 macrophages while suppressing pro-inflammatory responses [87].

In human monocyte-derived macrophages, omentin-1 promoted an anti-inflammatory M2 phenotype during monocyte-to-macrophage differentiation. Omentin-1 suppressed oxidized low-density lipoprotein (oxLDL)-induced foam cell formation by downregulating scavenger receptors (CD36, SR-A) and acyl-coenzyme A:cholesterol acyltransferase-1 (ACAT-1) (cholesterol esterification) while upregulating neutral cholesterol ester hydrolase 1 (NCEH1). In apolipoprotein E (*APOE*)^-/-^ mice, 4-week omentin-1 infusion retarded aortic atherosclerotic lesions with reduced macrophage/SMC content and inflammasome downregulation in peritoneal macrophages [93].

Unlike vaspin (which failed in HUVECs), omentin-1 has shown consistent anti-inflammatory effects in HUVECs. Omentin induced phosphorylation of AMPK and eNOS in HUVECs, increasing intracellular cGMP levels. Pretreatment with omentin significantly inhibited TNF-α-induced JNK phosphorylation and cyclooxygenase-2 (COX-2) expression. The mechanism was NO-dependent; a NOS inhibitor (L-NAME) reversed omentin’s inhibitory effect on TNF-α-induced COX-2, confirming the pathway. This represents the first demonstration that omentin plays an anti-inflammatory role in vascular endothelial cells [94].

Omentin-1 reduced THP-1 leukocyte attachment to HUVECs in a dose-dependent manner. Omentin-1 prevented oxLDL-induced expression of adhesion molecules VCAM-1 and E-selectin at both mRNA and protein levels. The mechanism involved restoration of Kruppel-like factor 2 (KLF2), a master transcriptional regulator of endothelial quiescence, which was suppressed by oxLDL. KLF2 restoration upregulated its target genes eNOS and PAI-1. KLF2 upregulation was mediated by p53, identifying a novel omentin-1/p53/KLF2/eNOS/adhesion molecule axis [95].

Recombinant omentin protein at physiological concentrations increased HUVEC differentiation into vascular-like structures (tube formation) and decreased apoptosis under serum starvation. Omentin stimulated phosphorylation of Akt and eNOS in HUVECs. Dominant-negative Akt or the PI3K inhibitor LY294002 blocked omentin’s effects on differentiation, survival, and eNOS phosphorylation, while dominant-negative AMPK diminished omentin-induced Akt phosphorylation. Critically, in eNOS-KO mice, systemic administration of omentin failed to improve blood flow in ischemic muscle, confirming eNOS as the essential in vivo effector [88].

Studies in HPMECs have demonstrated that omentin-1 protects against LPS-induced cytokine upregulation (IL-6, TNF-α) and NF-κB Rel subunit phosphorylation, with downstream preservation of endothelial barrier function assessed by transendothelial electrical resistance measurements [32].

In the cardiomyocyte models, omentin-1 has demonstrated robust anti-apoptotic and mitochondrial protective effects through multiple complementary mechanisms. Recombinant omentin suppressed H/R-induced apoptosis in neonatal rat cardiomyocytes by activating two independent pro-survival pathways, AMPK and Akt [96]. In H9C2 cardiomyoblasts, omentin pretreatment inhibited doxorubicin-induced apoptosis by suppressing mitochondrial ROS production, with the mitochondrial complex I inhibitor rotenone replicating omentin’s effects, confirming mitochondrial ROS as the critical upstream mediator [97]. Most comprehensively, in oxygen-glucose deprivation (OGD)-injured cardiomyocytes, omentin-1 enhanced mitochondrial accumulation of sirtuin 3 (SIRT3) and nuclear transduction of the forkhead box protein O3a (FOXO3a), restoring mitochondrial fusion–fission balance and activating PINK1/Parkin-dependent mitophagy to clear damaged mitochondria [98]. Together, these studies establish omentin-1 as a multi-level cardiomyocyte protectant that operates from the cell-surface (integrin-mediated Akt activation) through cytoplasmic kinase cascades (AMPK, Akt) to the mitochondrial level (ROS suppression, dynamics remodeling, and quality control via mitophagy).

In VSMCs, omentin-1 exerted anti-proliferative functions via AMPK/ERK suppression [99], and anti-migratory potential via NOX/ROS/p38/heat shock protein (HSP)27 suppression [100]. Omentin-1 suppressed angiotensin II-induced migration and PDGF-BB-induced proliferation in human aortic smooth muscle cells (HASMCs), and reduced collagen-1 and collagen-3 expression, showing anti-atherogenic effects [93].

Finally, in human periodontal ligament stem cells stimulated with LPS, recombinant omentin-1 reduced TNF-α, IL-1β, and IL-6 production and downregulated COX-2 and inducible (i)NOS expression, confirming consistent anti-inflammatory effects across diverse LPS-stimulated cell types [101].

A major limitation of the omentin-1 field is that no specific cell-surface receptor has been definitively identified. This contrasts sharply with chemerin (CMKLR1, GPR1, CCRL2) and vaspin (GRP78, LRP1).

In Table 5, the in vitro septic/inflammatory models studying omentin-1 are listed.

### 5.4. In Vivo Experimental Evidence

Omentin-1 has demonstrated consistent protective effects in murine models of lung injury and systemic inflammation. In an LPS-induced ALI murine model, omentin treatment suppressed pulmonary inflammation and preserved endothelial barrier function via an Akt/eNOS-dependent NO production, restoring VE-cadherin (adherens junctions) and F-actin cytoskeletal organization disrupted by LPS. Both prophylactic (adenoviral) and therapeutic (recombinant) omentin administration were effective, suggesting a potential therapeutic window even after ARDS onset. Mechanistically, the Akt/eNOS pathway enhanced endothelial NO production, contributing to preservation of vascular tone and barrier integrity, functions that are severely compromised in septic shock [32].

Omentin-1 expression was decreased in lungs of a bleomycin (BLM)-induced ALI model. Adenoviral overexpression of omentin-1 alleviated lung injury and maintained alveolar septa integrity. Omentin-1 overexpression reduced neutrophil and macrophage aggregation, decreased MCP-1 and IL-1β expression in lung tissue and suppressed oxidative stress and NF-κB activation in both in vivo lung tissue and in parallel LPS-stimulated macrophage experiments [102]. Another study demonstrated that omentin-1 can reverse established lung fibrosis in BLM-induced lung fibrosis by promoting mechanically activated myofibroblast dedifferentiation into lipofibroblasts [103].

Kataoka et al. provided the most comprehensive in vivo cardiac protection data using a murine myocardial I/R model [96]. They showed that systemic administration of human omentin to mice significantly reduced myocardial infarct size by enhancing phosphorylation of eNOS and suppressing phosphorylation of NF-κB in ischemic myocardium [96].

In a murine model of myocardial ischemia-induced HF, fat-specific AAV-omentin-1 overexpression ameliorated cardiac function, cardiac hypertrophy, infarct size, and cardiac pathological features in MI-induced HF mice. Omentin-1 enhanced SIRT3/FOXO3a signaling, increased mitochondrial fusion, decreased mitochondrial fission, and promoted PINK1/Parkin-dependent mitophagy. Additionally, circulating omentin-1 levels were diminished in HF patients compared to healthy subjects [98].

In a murine model of hindlimb ischemia, eNOS-dependent revascularization was demonstrated. Systemic adenoviral vector expressing omentin (Ad-omentin) delivery enhanced blood flow recovery and capillary density in ischemic limbs of wild-type mice, accompanied by increased Akt and eNOS phosphorylation. Critically, in *eNOS*-knockout mice, Ad-omentin failed to improve blood flow in ischemic muscle, confirming eNOS as the essential in vivo effector [88].

In cerebral ischemia, omentin-1 demonstrated neuroprotective effects. Lentiviral omentin-1 delivered 7 days before middle cerebral artery occlusion (MCAO) surgery significantly reduced brain infarction volume at 7 days post-injury. Moreover, CD34 and capillary density were increased in the cerebral ischemic penumbra, with enhanced eNOS and Akt phosphorylation and increased Bcl-2 expression [104].

Recombinant omentin-1 administered intra-peritoneally (IP) effectively ameliorated inflammation and repaired intestinal barrier in a murine dextran sulfate sodium (DSS)-induced colitis model. More specifically, omentin-1 decreased ROS and MDA levels, increased glutathione (GSH) and superoxide dismutase (SOD) production, and activated the Nrf2 pathway to regulate redox balance, ultimately protecting intestinal barrier function and reducing intestinal inflammation. The Nrf2 inhibitor ML385 partially reversed omentin-1’s protective effects, confirming Nrf2 dependence [105].

Intra-articular omentin-1 injection blocked collagen-induced arthritis by upregulating IL-4 and M2 macrophages while suppressing pro-inflammatory responses in a murine collagen-induced arthritis model [87].

In a murine atherosclerosis model (*APOE*-KO), 4-week omentin-1 infusion retarded aortic atherosclerotic lesions with reduced macrophage/SMC content and inflammasome downregulation in peritoneal macrophages [93].

Fat-specific omentin transgenic mice exhibited reduced neointimal thickening after arterial wire injury, with enhanced AMPK activation in injured arteries. AMPK inhibitor administration reversed the protection [99].

Subacute omentin-1 administration in normotensive rats significantly decreased mean blood pressure and pulse pressure without affecting heart rate or ECG. Omentin-1 increased plasma L-citrulline and adiponectin gene expression in pericardial adipose tissue, while decreasing *IL6* mRNA [106].

Finally, in a murine model of inflammation-induced osteoporosis, adenoviral delivery of omentin-1 significantly reduced systemic pro-inflammatory cytokine levels and protected against tissue damage [107]. This is considered relevant since systemic inflammatory states share cytokine-mediated pathways with sepsis.

Table 6 presents the findings from the omentin-1 studies of in vivo models of sepsis and organ injury.

Collectively, these in vivo data establish omentin-1 as capable of restraining organ-level inflammatory injury across multiple experimental models, with mechanistic pathways that are directly relevant to the organ dysfunction syndrome of clinical sepsis.

### 5.5. Clinical Evidence in Sepsis and Critical Illness

The largest clinical study for omentin-1 in sepsis is by Karampela et al., a prospective study of 102 septic patients versus 102 matched controls with serial sampling. As with chemerin, a parallel prognostic trend was observed for omentin-1 within the same cohort. Serum omentin-1 at admission effectively discriminated sepsis severity and predicted 28-day mortality with an AUC > 0.739. Admission levels of omentin-1 carried an independent mortality risk characterized by an HR of 2.26 (95% CI: 1.21–4.19, *p* = 0.01). Mirroring the chemerin kinetic profile, persistent elevation of omentin-1 at day 7 remained strongly associated with adverse 28-day outcomes, demonstrating an HR of 2.15 (95% CI: 1.43–3.22, *p* < 0.001). Notably, non-survivors failed to show the proportional physiological clearance or decline of these adipokines over the first week of ICU stay, establishing serial biomarker kinetics as an essential real-time window into organ dysfunction progression and ultimate ICU mortality. Omentin-1 correlated with APACHE II, SOFA, WBC count, and coagulation biomarkers, but notably not with procalcitonin, suggesting elevation through a pathophysiological axis distinct from the classical acute-phase response [23].

The landmark clinical study by Luedde et al. (*n* = 117 ICU patients, 84 septic, 33 non-septic; 50 healthy controls) provided the most comprehensive clinical data. Omentin-1 serum levels at ICU admission and after 72 h were not significantly different from those of healthy controls, a surprising finding given the preclinical data showing decreased omentin in ALI. Circulating omentin-1 levels were independent of sepsis etiology, since no difference was found between septic and non-septic critically ill patients. Omentin-1 was not associated with concentrations of inflammatory cytokines or routinely used sepsis markers (CRP, PCT), and moreover, omentin-1 was not predictive of short-term ICU survival. However, low omentin-1 concentrations were an independent predictor of overall (long-term) survival. Patients with lower omentin had better long-term outcomes. This counterintuitive finding (elevated omentin-1 predicting worse long-term survival) contrasts with preclinical data showing omentin-1 uniformly protective. The authors suggested this may reflect omentin’s role as an acute-phase reactant. This means its elevation in critical illness may represent a compensatory response by rising transiently to counteract inflammation, but persistent elevation signaling causes severe underlying metabolic derangement. Finally, omentin-1 levels strongly correlated with other adipokines (leptin receptor, adiponectin) that have also been identified as prognostic markers in critical illness, suggesting a coordinated adipokine response [84].

These findings are not necessarily contradictory. The Luedde cohort sampled a heterogeneous ICU population at admission, while the Karampela cohort specifically captured early sepsis. The kinetic trajectory of omentin, greater decline over the first week in survivors, appears to carry as much prognostic information as the absolute level, and may represent a measurable index of inflammatory resolution capacity [23,84].

In another study, circulating omentin-1 was decreased in patients with ARDS, correlating negatively with WBC and PCT, suggesting consumption at sites of endothelial injury or suppression by the acute inflammatory milieu. This finding is consistent with the general pattern that omentin-1 decreases in chronic inflammatory states but may behave differently in acute critical illness [32].

Similarly, Kukla et al. found omentin-1 was significantly decreased in COVID-19 patients, but this did not correlate with disease severity or ICU admission [19]. Wikar et al. found no difference in omentin-1 between COVID-19 patients and controls, with levels remaining stable over 7 days [63]. This inconsistency may reflect differences in disease severity (the Wikar cohort was predominantly non-critically ill), timing, or assay methodology.

These findings contrast with the elevation reported in the sepsis cohort of Karampela et al. and underscore that directionality is condition-specific, possibly reflecting differences in the dominant inflammatory mechanism (endothelial destruction in ARDS versus systemic immune dysregulation in sepsis) or timing relative to disease phase [23].

A comprehensive review by Watanabe et al. highlights a critical concept for interpreting omentin-1 in critical care as an acute-phase reactant exhibiting dual behavior. In chronic conditions (obesity, diabetes, metabolic syndrome), omentin-1 is consistently decreased and inversely associated with disease severity. However, in acute conditions (acute coronary syndrome, acute heart failure, acute inflammatory states), omentin-1 increases, acting as an acute-phase reactant with anti-inflammatory and atheroprotective effects. This dual behavior means that the interpretation of omentin-1 levels in sepsis depends critically on the timing of measurement relative to disease onset and the chronicity of the underlying condition [108]. The Karampela data [23] confirm that in acute sepsis, omentin-1 rises acutely and continues rising over the first week. Higher levels in non-survivors likely reflect greater inflammatory burden driving a stronger compensatory response rather than omentin-1 itself being harmful, analogous to how elevated cortisol in sepsis reflects stress response severity rather than cortisol toxicity.

## 6. Integrated Signaling Pathways

The three adipokines, chemerin, vaspin, and omentin-1, exert their effects in sepsis through distinct but converging molecular signaling pathways, with NF-κB serving as the central node, where their actions intersect. NF-κB activation is a central pathological event in sepsis, driving transcription of pro-inflammatory cytokines (TNF-α, IL-1β, IL-6, IL-8), adhesion molecules (ICAM-1, VCAM-1, E-selectin), and mediators of coagulation and endothelial dysfunction. Greater levels of nuclear NF-κB accumulation are associated with higher mortality and worse clinical outcomes in septic patients [109,110]. Importantly, all three adipokines reviewed here modulate NF-κB, but through distinct upstream mechanisms. Chemerin activates it via MAPK/ERK1/2 and PI3K/Akt, vaspin suppresses it via AMPK and KLK7, and omentin-1 suppresses it via TLR4/MyD88 inhibition and Nrf2 activation [28,30,42]. This mechanistic diversity is clinically important. It suggests that combination strategies targeting multiple adipokine pathways could provide additive benefit, and that the NF-κB-activating role of chemerin (pro-inflammatory in early infection) is balanced by the NF-κB-suppressing roles of vaspin and omentin-1 as counter-regulatory forces. The net effect on NF-κB activity in any given patient likely reflects the relative balance of these adipokines at any given disease stage.

**Figure 2:**
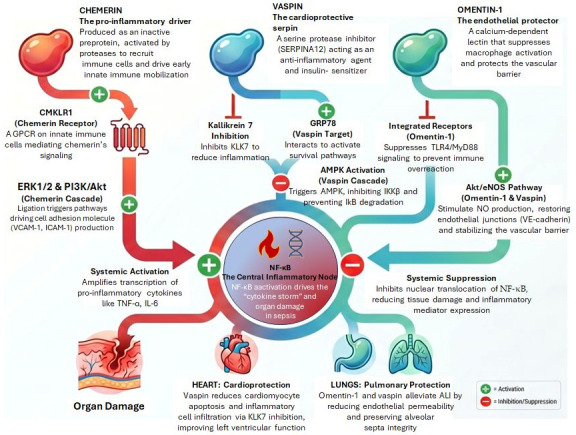
Integrated molecular signaling pathways converging on NF-κB in sepsis and organ injury. This figure illustrates the complex molecular “reprogramming” of the adipose–immune–metabolic axis, where NF-κB serves as the central node of convergence. During sepsis, dysfunctional adipose tissue releases specific adipokines that either amplify or suppress this central inflammatory driver. Chemerin (the pro-inflammatory driver): Chemerin signals through the G protein-coupled receptor CMKLR1 (ChemR23), activating the ERK1/2 MAPK and PI3K/Akt cascades. This leads to the systemic activation of NF-κB, which promotes the transcription of pro-inflammatory cytokines (e.g., TNF-α, IL-6) and adhesion molecules (e.g., ICAM-1, VCAM-1), ultimately driving leukocyte recruitment and endothelial organ damage. Vaspin (the cardioprotective serpin): Vaspin acts as a protective factor by inhibiting KLK7 and interacting with GRP78 to trigger survival pathways. It activates AMPK, which suppresses the IKK complex, thereby inhibiting NF-κB nuclear translocation. These actions provide cardioprotection by reducing cardiomyocyte apoptosis and inflammatory cell infiltration, and lung protection through the Akt/GSK-3β signaling pathway. Omentin-1 (the endothelial protector): Omentin-1 functions as a guardian of the vascular barrier by suppressing TLR4/MyD88 signaling, which prevents NF-κB overactivation. It further promotes systemic suppression of inflammation by activating the Akt/eNOS pathway, which stimulates NO production to restore endothelial adherens junctions (VE-cadherin) and preserve alveolar septa integrity in the lungs. The Central Node (NF-κB): NF-κB activation is the primary pathological event driving the “cytokine storm” and subsequent multi-organ dysfunction syndrome (MODS). The net clinical status of the patient reflects the competition between chemerin-driven activation (+) and the counter-regulatory suppression (–) provided by vaspin and omentin-1. Abbreviations: ALI, Acute lung injury; Akt, Protein kinase B; AMPK, Adenosine monophosphate-activated protein kinase; CMKLR1, Chemerin chemokine-like receptor 1; eNOS, Endothelial nitric oxide synthase; ERK1/2, Extracellular signal-regulated kinase 1/2; GSK-3β, Glycogen synthase kinase 3 beta; GRP78, Glucose-regulated protein 78; ICAM-1, Intercellular adhesion molecule 1; IKK, IkappaB kinase; IL-6, Interleukin 6; KLK7, Kallikrein-related peptidase 7; MAPK, Mitogen-activated protein kinase; MODS, Multi-organ dysfunction syndrome; MyD88, Myeloid differentiation primary response 88; NF-κB, Nuclear factor kappa-light-chain-enhancer of activated B cells; NO, Nitric oxide; PI3K, Phosphoinositide 3-kinase; TLR4, Toll-like receptor 4; TNF-α, Tumor necrosis factor alpha; VCAM-1, Vascular cell adhesion molecule 1; VE-cadherin, Vascular endothelial cadherin.

Table 7 presents the integrated signaling pathway summary for chemerin, vaspin, and omentin-1 in the context of sepsis and organ injury. Figure 2 illustrates how all three adipokines converge on NF-κB. The figure visually clarifies the specific receptors (CMKLR1, KLK7, GRP78) and the signaling cascades (ERK1/2, AMPK, Akt/eNOS) that the text describes in detail.

The convergence of these pathways on NF-κB has important therapeutic implications. NF-κB blockade in sepsis models corrects systemic hypotension, ameliorates myocardial dysfunction, inhibits pro-inflammatory gene expression, diminishes intravascular coagulation, reduces tissue neutrophil influx, and prevents microvascular endothelial leakage [110]. The anti-inflammatory adipokines vaspin and omentin-1 essentially mimic aspects of NF-κB inhibition through endogenous, physiologic mechanisms. Vaspin via AMPK-mediated IKK suppression and omentin-1 via TLR4/MyD88 blockade and Nrf2 counter-regulation. Chemerin, conversely, amplifies NF-κB activation through MAPK/PI3K-Akt, though its receptor CMKLR1 can paradoxically promote inflammation resolution when engaged by alternative ligands like RvE1.

## 7. Aggregation and Evaluation of Preclinical/Clinical Data

Table 8, Table 9 and Table 10 summarize the findings of all three adipokines in septic/inflammatory/ALI animal models, their evidence in sepsis/critical illness, and COVID-19 clinical studies, respectively.

A major conceptual challenge highlighted by this comprehensive review is the stark discrepancy between the uniform organ-protective effects of vaspin and omentin-1 observed in preclinical animal models and their direct association with adverse clinical outcomes and increased 28-day mortality in septic patients. Mechanistically, in vitro and in vivo models depict these novel adipokines as potent counter-regulatory molecules that suppress the NF-κB axis, preserve endothelial barrier integrity, and alleviate acute lung injury. However, clinical data consistently reveal that higher circulating concentrations serve as indicators of poor prognosis. To reconcile this paradox, these adipokines must be critically evaluated not as primary pathogenic mediators of injury but rather as compensatory acute-phase reactants or sensitive biomarkers of underlying tissue stress and disease severity. Much like the classic elevation of endogenous cortisol or anti-inflammatory cytokines (e.g., IL-10) during severe septic shock, the hypersecretion of omentin-1 and chemerin into the circulation likely represents a maximal, systemic attempt by the host’s “adipose–immune–metabolic axis” to mitigate overwhelming inflammation and secure metabolic homeostasis. Therefore, their concentration in human plasma directly mirrors the magnitude of the infectious insult; non-survivors exhibit significantly higher levels because they are experiencing a greater degree of systemic tissue damage and multi-organ failure, which continuously drives compensatory adipokine production.

Beyond baseline values at ICU admission, the evaluation of serial biomarker kinetics provides critical insights into the progression of organ dysfunction. The striking observation that a failure to clear chemerin or omentin-1 over the first week of sepsis results in a steep rise of hazard ratios (HRs of 10.01 and 2.15, respectively) demonstrates that tracking temporal trajectories is far superior to single-point sampling. Persistent elevation acts as a real-time clinical proxy for a “failed resolution” phenotype, signaling that the underlying microvascular beds and visceral adipose depots remain locked in a hyper-inflammatory loop.

However, several formidable translational challenges currently limit the clinical implementation of these molecules. First, there is a distinct lack of humanized preclinical validation; rodent models of sepsis fail to mimic human comorbidities, baseline adipose volumes, or the typical delayed healthcare timelines seen in real-world ICU environments. Second, extreme clinical heterogeneity, confounded by BMI variations, fluid resuscitation-induced pseudo-obesity, and varying degrees of acute kidney or liver clearance capacity, makes establishing uniform diagnostic or prognostic cut-off values exceptionally difficult. Until standardized, high-throughput clinical assays are validated across multicenter cohorts to isolate specific active adipokine isoforms from their inactive pro-forms, these molecules will remain highly informative pathophysiological mirrors rather than ready-to-use clinical tools.

**Figure 3:**
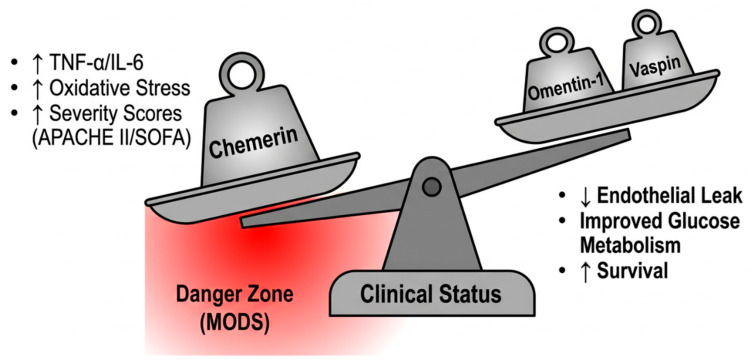
The adipokine balance in sepsis. The “see-saw” model of adipokine dysregulation, illustrating the shift from homeostasis toward septic shock and MODS. Label “Clinical Status”: represents the patient’s physiological stability. The pro-inflammatory tray (tilted downwards): Driven by a high concentration of chemerin, this side represents clinical deterioration. Associated effects include a surge in pro-inflammatory cytokines (TNF-α, IL-6), increased oxidative stress, and a direct correlation with rising APACHE II and SOFA severity scores. The underlying “Danger Zone” gradient signifies the high risk of MODS. The protective tray (tilted upwards): Represented by omentin-1 and vaspin, these adipokines act as counter-regulatory weights. Their biological presence is associated with endothelial barrier integrity, improved glucose metabolism, and increased 28-day survival. The balance between these opposing forces dictates the trajectory of critical illness; a persistent tilt toward the chemerin-heavy side is a hallmark of poor prognosis. Abbreviations: APACHE II, Acute physiology and chronic health evaluation II; IL-6, Interleukin 6; MODS, Multiple organ dysfunction syndrome; SOFA, Sequential organ failure assessment; TNF-α, Tumor necrosis factor alpha. An upward arrow indicates an increase; a downward arrow indicates a decrease.

Figure 3 depicts the adipokine balance in sepsis. It is a synthesis of the clinical and prognostic data. It illustrates the shift from homeostasis to MODS. As seen in the figure, the patient’s survival depends on the balance between pro-inflammatory drivers (chemerin) and protective factors (vaspin and omentin-1).

As mentioned above, a critical methodological limitation across the clinical studies reviewed is the inconsistent measurement and adjustment for body composition. Most studies used BMI as the sole anthropometric measure. Yet, BMI is a particularly poor surrogate for adiposity in critically ill patients due to fluid overload-induced pseudo-obesity, inability to distinguish fat from lean mass, and failure to capture fat distribution [12]. This limitation is especially consequential for chemerin, vaspin, and omentin-1, as all three are depot-specific adipokines preferentially produced by visceral adipose tissue (VAT). Chemerin expression in visceral, but not subcutaneous adipose tissue (SAT), drives its elevated systemic levels in obesity, while vaspin and omentin-1 are produced almost exclusively by visceral (omental) adipose tissue [81]. The VAT-to-SAT ratio, measurable from routine abdominal CT scans, is uncorrelated with BMI yet independently predictive of 90-day mortality in sepsis, with higher ratios associated with a more pro-inflammatory cytokine profile [111]. Future studies investigating these adipokines in sepsis should include waist circumference alongside BMI and ideally incorporate CT-derived visceral and subcutaneous adipose tissue quantification. Such measures would allow investigators to unravel whether altered adipokine levels in sepsis reflect the acute inflammatory response, baseline visceral adiposity, or a combination of both, and would provide the granularity needed to test whether these adipokines mediate the relationship between fat distribution and sepsis outcomes.

## 8. Shared Pathophysiological Mechanisms

### 8.1. Universal Elevation as an Acute-Phase Phenomenon

All three adipokines are elevated in sepsis relative to healthy controls, distinguishing them from classic anti-inflammatory adipokines such as adiponectin, which typically falls in critical illness [21,23,83]. This acute-phase elevation likely reflects a combination of increased synthesis driven by inflammatory cytokines (TNF-α, IL-6), release from catabolizing adipose tissue, and potentially reduced clearance secondary to hepatic or renal dysfunction. Whether the elevation represents a protective adaptive response or a pathological process remains an open question.

### 8.2. Kinetics as Prognostic Signal

For both chemerin and omentin-1, the trajectory of change over the first week of sepsis carries significant independent prognostic information [21,23]. Non-survivors show not only higher initial levels but a markedly attenuated decline, suggesting that the failure to resolve the acute adipokine response mirrors the failure to resolve the underlying inflammatory state. This kinetic approach to biomarker interpretation represents a conceptual advance beyond single time-point measurements and may be particularly valuable in guiding treatment decisions.

### 8.3. Convergence of Experimental and Clinical Evidence

A notable strength of the evidence base for these three adipokines is the concordance between experimental and clinical findings. Vaspin’s cardioprotective effects demonstrated in CLP murine models [34] are supported by elevated levels in human sepsis [83]. Omentin-1’s anti-inflammatory and lung-protective effects in murine ALI models are consistent with its elevation and prognostic utility in human sepsis [32]. Chemerin’s dual pro- and anti-inflammatory roles in experimental models [35,36,43] are reflected in its complex clinical biomarker profile, where kinetics matter as much as absolute levels [23].

Table S1 provides a structured comparison of the key characteristics of chemerin, vaspin, and omentin-1 in critical illness and sepsis, integrating experimental and clinical data.

### 8.4. Intersection of Metabolism and Immunity

All three adipokines participate in both metabolic and immune regulation, simultaneously regulating insulin sensitivity, glucose metabolism, and systemic inflammation [6,39,60,65]. This dual role is particularly relevant in critical illness, where insulin resistance, hyperglycemia, dyslipidemia, and immune dysregulation co-exist and mutually amplify organ injury. Novel adipokines may thus offer a “metabolic–immune” biomarker profile that conventional cytokine or acute-phase measurements cannot capture.

### 8.5. Therapeutic Potential

Beyond their roles as biomarkers, the biological functions of these adipokines represent mechanistically tractable therapeutic targets. The therapeutic potential of these adipokines is supported exclusively by preclinical data and remains entirely speculative for human sepsis. Nevertheless, several features make them attractive therapeutic candidates.

Both omentin-1 and vaspin have been successfully administered as recombinant proteins in animal models with therapeutic efficacy. Qi et al. demonstrated that a single shot of recombinant human omentin administered after LPS challenge protected against established ARDS, a critical finding for clinical translatability, as most sepsis patients present after disease onset [32,33].

Chemerin-derived peptides offer pharmacological advantages. The synthetic peptide C15 demonstrated anti-inflammatory efficacy at extraordinarily low doses (0.32 ng/kg), suggesting high potency and potentially favorable pharmacokinetics [52].

All three adipokines converge on AMPK, NF-κB, and PI3K/Akt pathways that are already being targeted by other therapeutic strategies in sepsis. Vaspin’s cardioprotection via KLK7 inhibition [34] and endothelial protection via AMPK/NF-κB [28] are sufficiently characterized in experimental models to warrant consideration for therapeutic development. Chemerin’s resolution-promoting activity via CMKLR1 agonism, supported by recent cryo-EM structural data, offers a promising target for pro-resolving pharmacology [35]. Omentin-1’s endothelial and pulmonary protection via Akt/eNOS and NF-κB pathways [32] provides multiple therapeutic entry points for sepsis-associated organ injury. This raises the possibility of pharmacological adipokine modulation using existing drugs rather than recombinant proteins.

No adipokine-targeted therapy has reached clinical application in sepsis, but several approaches are under investigation. Adiponectin mimics (AdipoRon) and glucagon-like peptide-1 (GLP-1) receptor agonists are explored as potential organ-protective agents [18]. The key challenge remains that adipokines function as a network rather than individually, and their effects are stage-dependent; what is protective in one phase may be harmful in another.

## 9. The Broader Adipokine Network in Sepsis

Chemerin, vaspin, and omentin-1 operate within a broader adipokine network whose collective dynamics shape the host response in critical illness. Understanding their behavior relative to resistin, adiponectin, leptin, and extracellular nicotinamide phosphoribosyltransferase (eNampt) is essential for contextualizing their clinical utility.

### 9.1. Resistin

Among all adipokines studied in sepsis, resistin is most tightly integrated into the core inflammatory cytokine network. Hierarchical clustering analysis demonstrated that resistin clusters with IL-6, IL-8, MCP-1, and IL-10 on days 1, 2, and 4 of sepsis, forming a prognostic-related network significantly associated with SOFA scores, disseminated intravascular coagulation (DIC) scores, and mortality [17]. Notably, chemerin and vaspin did not cluster within this core network, suggesting they reflect parallel but partially independent immunometabolic pathways [17]. Resistin is markedly elevated in sepsis (approx. 6-fold above controls), correlates with APACHE II and SOFA, and is highest in patients with the hyperinflammatory immunological endotype, independent of BMI [14]. Sustained elevation of resistin alongside eNampt/visfatin independently predicts 28-day mortality [112].

### 9.2. Adiponectin

Adiponectin exerts anti-inflammatory, anti-diabetic, and anti-atherogenic effects via the adiponectin receptors (AdipoR), AdipoR1, AdipoR2, and T-cadherin [18]. In critical illness, adiponectin levels are initially low and gradually rise during recovery, creating a reciprocal temporal pattern to resistin that may reflect a biological shift from the hyperacute pro-inflammatory to the reparative phase [113]. A gradual rise in adiponectin is associated with better outcomes, while sustained low levels may indicate failure of the anti-inflammatory recovery program [113]. GLP-1 receptor agonists represent a promising therapeutic strategy to boost adiponectin levels and link metabolic therapies to sepsis immunomodulation [18].

### 9.3. Leptin, eNampt/Visfatin

Leptin shows inconsistent associations with sepsis outcomes, with circulating levels higher in obese septic patients but not independently associated with immunological endotypes or 28-day mortality, suggesting it reflects baseline adiposity more than the acute response [14,114]. eNampt/visfatin, a pro-inflammatory adipokine, correlates strongly with resistin in the acute phase and, when both are sustained, independently predicts 28-day mortality [112].

Table S2 lists the findings from the broader adipokine network in sepsis. These adipokine dynamics may partly explain the “obesity paradox”, though a study of 167 septic patients found the relationship between resistin and mortality was independent of BMI, suggesting the paradox may be mediated through mechanisms beyond circulating adipokine levels, such as greater energy reserves and adipose tissue morphologic adaptations including M2 macrophage phenotypic switching [14].

## 10. Clinical Insights

The evidence for chemerin, vaspin, and omentin-1 in sepsis follows a consistent pattern. Robust preclinical mechanistic data contrasted with limited and sometimes contradictory clinical findings. This preclinical–clinical disconnect is not unique to these adipokines; it mirrors the broader failure of sepsis immunotherapy, where over 100 clinical trials targeting the immune-inflammatory cascade have failed despite compelling preclinical rationale [115,116]. The reasons for this disconnect are multifactorial and specific to each adipokine.

For chemerin, the preclinical data demonstrate a predominantly anti-inflammatory role via ChemR23-dependent pDC recruitment, enhanced phagocytosis, and efferocytosis. Yet clinical data consistently show that higher chemerin levels predict worse outcomes [21]. This apparent paradox can be reconciled by recognizing that elevated circulating chemerin in sepsis likely reflects reactive upregulation, a compensatory response to overwhelming inflammation rather than a pathogenic driver. The tissue–circulation discordance observed by Horn et al., i.e., elevated circulating chemerin but decreased VAT mRNA expression, supports this interpretation, suggesting that chemerin is being proteolytically activated and released from tissue stores rather than newly synthesized [60]. Whether this compensatory response is sufficient, insufficient, or ultimately maladaptive remains unknown.

Chemerin emerges as the most clinically promising biomarker among the three, with independent prognostic value for 28-day mortality in sepsis and COVID-19 ARDS. Its kinetics (sustained elevation leading to worse prognosis) add dynamic prognostic information beyond single time-point measurements. However, the contradictory direction of change across COVID-19 studies (elevated in some, decreased in others) and the context-dependent mortality relationship (opposite in SHG vs. non-SHG patients) complicate clinical interpretation [19,21,60,62].

Vaspin has a critical translational gap, despite strong preclinical evidence of organ protection in sepsis models (reduced mortality in CLP via KLK7 inhibition). Only a small dedicated clinical sepsis study has been conducted, demonstrating increased vaspin in septic patients compared to the control group of critically ill patients receiving intensive care after trauma or major surgery [83]. The single COVID-19 study measuring vaspin found no change, but this was performed in a relatively small cohort [19].

For omentin-1, the same paradox as in chemerin applies. Preclinical data uniformly show organ protection (ALI, cardiac I/R, lung fibrosis), yet the Karampela prospective study found that higher omentin-1 independently predicted 28-day mortality [23]. The concept of omentin as an acute-phase reactant, rising in response to acute inflammatory stress, provides the most parsimonious explanation. Elevated omentin-1 in non-survivors likely reflects the magnitude of the inflammatory insult driving the compensatory response, not a harmful effect of omentin itself. This interpretation is supported by the observation that omentin’s kinetics (rising trajectory over the first week) are opposite to chemerin’s (declining trajectory). Yet both show the same prognostic pattern that sustained elevation predicts mortality.

Omentin-1 has comparable-quality prospective evidence with chemerin, establishing it as an independent predictor of 28-day mortality. Its opposite kinetic trajectory to chemerin (rising vs. declining) makes it a potentially valuable complementary biomarker [21,23]. However, it presents a paradox; while preclinical data uniformly show protective effects, one ICU clinical study found that elevated omentin predicted worse long-term survival, and another that it increases in sepsis, and higher levels and lower kinetics during the first week of sepsis are associated with severity and 28-day mortality [23,84]. This may reflect omentin’s dual role as both a protective adipokine and an acute-phase reactant. Omentin-1 elevation in sepsis may indicate ongoing compensatory stress, rising acutely and continuing to rise, with higher levels reflecting greater disease severity rather than a protective effect.

The disconnect between preclinical promise and clinical complexity underscores the need for larger, longitudinal studies with serial measurements and phenotypic stratification [108]. A fundamental limitation across all three adipokines is the absence of interventional clinical trials. All human data are observational, making it impossible to determine whether altered adipokine levels are causative, compensatory, or merely epiphenomenal in sepsis pathophysiology [15,16].

## 11. Limitations and Future Directions

The evidence base reviewed here, while promising, faces important limitations.

In the experimental domain, most in vitro studies use single cell types stimulated with LPS, a model that, while useful, does not recapitulate the complexity of polymicrobial human sepsis [31]. The minimum quality threshold in pre-clinical sepsis studies (MQTiPSS) consensus guidelines explicitly state that “endotoxin injection should not be considered as a model of sepsis”. LPS produces an acute, transient cytokine storm with hypodynamic hemodynamics, whereas human sepsis involves living bacteria, sustained low-level cytokine release, and hyperdynamic hemodynamics. Several key studies for vaspin and omentin-1 used LPS-ARDS models, and their findings may not translate to polymicrobial sepsis [117].

In vivo murine models, including CLP, recapitulate key features of human sepsis but differ substantially in immune response kinetics, adipose tissue biology, and pharmacokinetics of recombinant adipokines. While CLP is considered the gold standard, it produces a polymicrobial peritonitis that may not reflect the most common sepsis etiologies (pneumonia, urinary tract infection). The CLP model also lacks the comorbidities (diabetes, obesity, advanced age) that characterize most sepsis patients. Many preclinical studies administered adipokines before the septic insult (prophylactic design), which has no clinical relevance. The Qi et al. study is notable for including both prophylactic and therapeutic arms, the latter being far more clinically relevant [32]. Murine adipose tissue differs substantially from human adipose tissue in depot distribution, adipokine expression profiles, and immune cell composition. The MQTiPSS guidelines emphasize that “testing beyond rodent models is especially crucial” for translational relevance [115].

The study by Mohlis et al. on vaspin is the most recent published work in this field and illustrates that the mechanistic landscape continues to evolve rapidly [67]. Translation from mouse to human remains a significant challenge for this field, as for all sepsis therapeutics.

In the clinical domain, most studies are single-center cohort studies with 50–120 patients [21,23,83,84]. The influence of major confounders including obesity, diabetes mellitus, chronic liver disease, and renal impairment must be systematically accounted for, as demonstrated by the confounding effect of liver cirrhosis on chemerin levels [61] and the effect of renal function on vaspin clearance [68]. The condition-specific directionality of omentin-1 (elevated in sepsis, depleted in ARDS and COVID-19) requires reconciliation through studies with detailed, high-resolution analysis of specific clinical, molecular, or imaging traits, ideally with concurrent BAL and serum sampling [19,23,32]. Additionally, the relationship between circulating levels and tissue-level activity is discordant for chemerin (elevated in serum but decreased in visceral adipose tissue mRNA), raising questions about the source and functional significance of circulating levels [60]. Whether adipokine-guided risk stratification adds incremental value beyond existing biomarkers (PCT, lactate, SOFA) is unknown. The stage-dependent behavior of these adipokines, the same molecule may be protective in one phase and harmful in another, complicates therapeutic targeting.

Perhaps the most fundamental unresolved question is whether altered adipokine levels in sepsis are causative (driving pathology), compensatory (protective responses to injury), or epiphenomenal (bystander markers of disease severity). Alipoor et al. explicitly identified this as the central knowledge gap: “further studies are required to clarify whether the reason for these changes is pathophysiological or compensatory” [16].

Nutritional interventions may also modulate adipokine profiles. Different parenteral lipid emulsions differentially affect adipokine trajectories [118]. This suggests that nutritional support strategies could indirectly influence adipokine-mediated outcomes. The mechanistic link between elevated adipokine levels and mortality, whether causal or epiphenomenal, also remains to be established. Multicenter validation studies using standardized Sepsis-3 criteria [1] and harmonized assay platforms are urgently needed. Most available assays are research-grade ELISAs not validated for clinical use, with significant inter-assay variability.

BMI confounding remains unresolved. Most studies did not adequately adjust for body composition, and the relationship between adipokine levels and outcomes could be confounded by adiposity rather than reflecting sepsis-specific pathophysiology.

The concept of endotype-specific biomarker utility has not been explored. Recent advances in sepsis precision medicine have identified reproducible molecular endotypes (hyperinflammatory vs. immunoparalytic) that respond differently to treatment. Whether chemerin, vaspin, or omentin-1 levels differ across these endotypes and whether their prognostic value is endotype-dependent is entirely unknown. The de Nooijer et al. finding that resistin’s association with outcomes was driven by the hyperinflammatory endotype, not by obesity, provides a template for how such analyses should be conducted for these novel adipokines [14,116,119].

Future research priorities include: (1) large multicenter prospective studies with serial sampling, (2) clinical-grade validated assays, (3) isoform-specific measurements, (4) comparison with established biomarkers, (5) mechanistic studies in humanized animal models, and (6) early-phase clinical trials targeting CMKLR1, KLK7, and omentin-1 pathways. No adipokine-targeted therapy has reached clinical application in sepsis, and all remain investigational.

## 12. Conclusions

In conclusion, chemerin, vaspin, and omentin-1 represent a coherent group of novel adipokines with complementary biological functions, convergent signaling pathways, and emerging clinical relevance in sepsis and acute lung inflammation. The preclinical evidence consistently demonstrates organ-protective effects mediated through AMPK, NF-κB, PI3K/Akt, and NLRP3 inflammasome pathways. Clinical data, strongest for chemerin and omentin-1, establish both as independent predictors of 28-day mortality in sepsis, with opposite but complementary kinetic trajectories that may provide dynamic prognostic information. However, the field faces several critical challenges: the absence of clinical data for vaspin in sepsis, inadequate body composition phenotyping across studies, the unresolved causation–compensation–epiphenomenon question, and the complete absence of interventional trials. Future research should prioritize prospective multi-adipokine studies with CT-derived body composition assessment, Mendelian randomization to establish causality, and integration of adipokine profiling into the emerging precision medicine framework for sepsis. The convergence of adipokine biology with the adipose–immune–metabolic axis concept and the disease tolerance paradigm offers a compelling theoretical foundation for developing novel, tolerance-based therapeutic strategies that could complement existing pathogen-targeted and immunomodulatory approaches in sepsis.

## Figures and Tables

**Table 1:** Chemerin in in vitro septic/inflammatory models.

Cell Type	Model	Chemerin Effect	Mechanism	Key Findings	References
HUVECs	Chemerin	Pro-inflammatory	ERK1/2, PI3K/Akt → NF-κB	↑ E-selectin, VCAM-1, ICAM-1; ↑ monocyte adhesion	[42]
HUVECs	TNF-α+ chemerin pretreatment	Anti-inflammatory	Akt/eNOS → NO → NF-κB/p38 suppression	↓ VCAM-1, ↓ monocyte adhesion (NO-dependent)	[44]
HMVECs, VSMCs	Chemerin	Pro-inflammatory, proliferative	NOX → ROS → MAPK	↑ ROS, ↑ proliferation, ↓ eNOS/NO, ↑ apoptosis	[45]
Mouse/human macrophages	LPS ± IFN-γ + chemerin	No effect	N/A	No change in TNF-α, IL-1β, IL-6, IL-10	[46]
Mouse peritoneal macrophages	Chemerin	Pro-adhesive	Gαi→PI3K/Akt/p38 → integrin clustering	Rapid adhesion to fibronectin (VLA-5) and VCAM-1 (VLA-4)	[48]
Endothelial cells	LPS/TNF-α/IL-1β	CCRL2 upregulation	NF-κB, JAK/STAT	CCRL2 presents chemerin to CMKLR1+ cells; ↑ NK adhesion	[49]
Inflammatory macrophages	Chemerin	Regulated by GRK6/β-arrestin 2	Receptor desensitization	GRK6/β-arrestin 2 deficiency → ↑ migration, altered Akt/ERK	[41]
Peritoneal macrophages	TLR ligands, cytokines	CMKLR1 regulation	TLR/cytokine signaling	Pro-inflammatory stimuli ↓ CMKLR1; TGF-β ↑ CMKLR1	[50]

Abbreviations: β-arrestin 2: Beta-arrestin 2; CCRL2: C-C Motif chemokine receptor-Like 2; CMKLR1: Chemokine-like receptor 1; eNOS: Endothelial nitric oxide synthase; ERK1/2: Extracellular signal-regulated kinase 1/2; Gαi: G protein subunit alpha i; GRK6: G protein-coupled receptor kinase 6; HMVECs: Human microvascular endothelial cells; HUVECs: Human umbilical vein endothelial cells; ICAM-1: Intercellular adhesion molecule-1; IFN-γ: Interferon-gamma; IL-1β: Interleukin-1 beta; IL-6: Interleukin-6; IL-10: Interleukin-10; JAK: Janus kinase; LPS: Lipopolysaccharide; MAPK: Mitogen-activated protein kinase; NF-κB: Nuclear factor-kappa B; NK: Natural killer cell; NO: Nitric oxide; NOX: NADPH oxidase; p38: p38 Mitogen-activated protein kinase; PI3K/Akt: Phosphoinositide 3-kinase/Protein kinase B; ROS: Reactive oxygen species; STAT: Signal transducer and activator of transcription; TGF-β: Transforming growth factor-beta; TNF-α: Tumor necrosis factor-alpha; VCAM-1: Vascular cell adhesion molecule-1; VLA-4/VLA-5: Very late antigen-4/5; VSMCs: Vascular smooth muscle cells. An upward arrow indicates an increase; a downward arrow indicates a decrease.

**Table 2:** Chemerin in vivo models of sepsis and organ injury.

Model	Species	Intervention	Key Findings	Mechanism	References
LPS-induced ALI	Mouse	Exogenous chemerin; *CMKLR1*-KO	Chemerin ↓ neutrophil infiltration, ↓ cytokines; *CMKLR1*-KO mice ↑ neutrophils	pDC recruitment via ChemR23	[47]
Zymosan peritonitis	Mouse	C15 peptide (0.32 ng/kg); *CMKLR1*-KO	C15 ↓ neutrophils 63%, ↓ monocytes 62%; no effect in KO; anti-chemerin Ab ↑ inflammation	ChemR23-dependent	[52]
Zymosan/thioglycollate peritonitis	Mouse	C15 (8 pg/mouse)	C15 ↑ phagocytosis, ↑ efferocytosis; ↓ apoptotic/necrotic cells; impaired in ChemR23-KO	ChemR23/Syk → actin polymerization	[53]
Zymosan/thioglycollate peritonitis	Mouse	*CCRL2*-KO	↑ Myeloid recruitment, ↑ chemerin/CXCL1; anti-chemerin Ab reversed phenotype	Unregulated chemerin bioavailability	[54]
LPS-induced ALI	Mouse	*CCRL2*-KO	↓ CMKLR1^+^ NK cell recruitment to airways; ↑ plasma chemerin	Loss of endothelial chemerin presentation	[49]
Peritoneal sepsis (CLP-like)	Mouse	Translational model	↑ Circulating chemerin; ↓ VAT mRNA; chemerin correlates with severity	Tissue–circulation discordance	[60]
LPS-induced ALI	Mouse	scRNA-seq; chemerin neutralization	rM-ed neutrophils ↑ CCRL2; chemerin neutralization ↓ reverse migration	CCRL2-mediated neutrophil reverse migration	[56]
LPS-induced ARDS	Rat	RvE1 (10 μg/kg IV, post-LPS)	↑ Survival (30→70%); ↑ alveolar fluid clearance; ↓ lung injury	PI3K/AKT/SGK1 → ENaC/NKA ↑	[57]
Bacterial pneumonia (*E. coli*)	Mouse	RvE1 (0.005 mg/kg IV)	↓ Neutrophils 55%; ↑ bacterial clearance; ↑ survival	↓ IL-1β, IL-6, HMGB1, chemokines	[58]
Pulmonary inflammation (*E. coli*, carrageenan)	Mouse	RvE1	↑ Neutrophil apoptosis; ↑ macrophage efferocytosis; resolution of ALI	BLT1 → NADPH oxidase → caspase-8/3	[59]

Abbreviations: Ab: Antibody; AKT: Protein kinase B; ALI: Acute lung injury; ARDS: Acute respiratory distress syndrome; BLT1: Leukotriene B4 receptor 1; C15: Chemerin15 peptide; CCRL2: C-C motif chemokine receptor-like 2; ChemR23: Chemerin receptor 23; CLP: Cecal ligation and puncture; CMKLR1: Chemokine-like receptor 1; CXCL1: C-X-C motif chemokine ligand 1; ENaC: Epithelial sodium channel; HMGB1: High mobility group box 1; IL-1β: Interleukin-1 beta; IL-6: Interleukin-6; IV: Intravenous; KO: Knockout; LPS: Lipopolysaccharide; NADPH: Nicotinamide adenine dinucleotide phosphate; NKA: Na^+^/K^+^-ATPase; pDC: Plasmacytoid dendritic cell; PI3K: Phosphoinositide 3-kinase; rM-ed: Reverse-migrated; RvE1: Resolvin E1; scRNA-seq: Single-cell RNA sequencing; SGK1: Serum/Glucocorticoid-regulated kinase 1; Syk: Spleen tyrosine kinase; VAT: Visceral adipose tissue. An upward arrow indicates an increase; a downward arrow indicates a decrease.

**Table 3:** Vaspin in in vitro septic/inflammatory models.

Cell Type	Stimulus	Vaspin Effect	Mechanism	Key Outcome	References
HAECs	TNF-α	Anti-inflammatory	AMPK → NF-κB ↓	↓ ICAM-1, VCAM-1, E-selectin, MCP-1; ↓ monocyte adhesion	[28]
EA.hy926	TNF-α, IL-1	Anti-inflammatory	NF-κB ↓ (dose-dependent)	↓ TNF-α, IL-1, IL-6; ↓ ICAM-1, VCAM-1, MCP-1	[69]
HUVECs	TNF-α	No effect	N/A	No change in JNK, p38, NF-κB, adhesion molecules	[70]
HPMECs	LPS	Anti-inflammatory, anti-apoptotic	Akt/GSK-3β → NF-κB ↓, NADPH oxidase ↓	↓ TNF-α, IL-6; ↓ ROS; ↓ apoptosis; no AJ change	[33]
Rat VSMCs	TNF-α	Anti-inflammatory	ROS ↓ → NF-κB/PKCθ ↓	↓ ICAM-1; ↓ monocyte adhesion	[71]
H9C2 cardiomyocytes	TNF-α	Anti-apoptotic	PI3K/Akt/mTOR ↓ → autophagy ↑	↑ LC3-II/I, Beclin-1; ↓ apoptosis	[72]
H9C2 cardiomyocytes	H/R	Anti-apoptotic	AMPK-mTOR → autophagic flux ↑	↓ Apoptosis (chloroquine-reversible)	[73]
H9C2 cardiomyocytes	H/R	Anti-inflammatory	TLR4 ↓ → NF-κB ↓	↓ IL-1β, IL-18, TNF-α (dose-dependent)	[74]
H9C2 cardiomyocytes	High glucose	Anti-inflammatory	Autophagy ↑ → NLRP3 ↓	↓ Caspase-1, IL-1β, TNF-α; ↓ mito ROS	[75]
3T3-L1 adipocytes	IL-1β	Anti-inflammatory	IKKα/β → IκB → NF-κB ↓	↓ IL-6, MCP-1, TNF-α; ↑ insulin-stimulated pAkt	[29]
HK-2 renal cells	H/R	Anti-inflammatory, anti-ER stress	HMGB1 ↓ → Nrf2/HO-1 ↑, NF-κB ↓	↓ GRP78, ATF6, CHOP; ↓ inflammation	[76]
Hepatocytes	ER stress	Metabolic protection	GRP78/MTJ-1 → pAkt ↑, pAMPK ↑	↓ ER stress markers; improved glucose tolerance	[77]
HAECs	ER stress/diabetic milieu	Anti-apoptotic	GRP78/VDAC → pAkt ↑	↓ Ca^2+^ influx; ↓ apoptosis; Kd = 0.565 nM	[78]

Abbreviations: 3T3-L1: Adipocyte cell line; AJ: Adherens junction; AMPK: Adenosine monophosphate-activated protein kinase; ATF6: Activating transcription factor 6; CHOP: CCAAT-enhancer-binding protein homologous protein; COX-2: Cyclooxygenase-2; EA.hy926: Human endothelial cell line; ER: Endoplasmic reticulum; GRP78: Glucose-regulated protein 78 kDa; H9C2: Rat cardiomyoblast cell line; HAECs: Human aortic endothelial cells; HK-2: Human kidney-2 renal cell line; HMGB1: High mobility group box 1; HO-1: Heme oxygenase-1; HPMECs: Human pulmonary microvascular endothelial cells; HUVECs: Human umbilical vein endothelial cells; H/R: Hypoxia/Reoxygenation; ICAM-1: Intercellular adhesion molecule-1; IκB: Inhibitor of kappa B; IKKα/β: IkappaB Kinase alpha/beta; IL-1/IL-1β: Interleukin-1/Interleukin-1 beta; IL-6: Interleukin-6; IL-18: Interleukin-18; JNK: c-Jun N-terminal kinase; Kd: Dissociation constant; LC3-II/I: Microtubule-associated protein 1A/1B-light chain 3; LPS: Lipopolysaccharide; MCP-1: Monocyte chemoattractant protein-1; mTOR: Mammalian target of rapamycin; MTJ-1: Murine DNAJ homolog 1; NF-κB: Nuclear factor-kappa B; NLRP3: NOD-, LRR- and pyrin domain-containing protein 3; Nrf2: Nuclear factor erythroid 2-related factor 2; p38: p38 Mitogen-activated protein kinase; pAkt: phosphorylated Akt; PI3K/Akt: Phosphoinositide 3-kinase/Protein kinase B; PKCθ: Protein kinase C theta; ROS: Reactive oxygen species; siRNA: small interfering RNA; TNF-α: Tumor necrosis factor-alpha; VCAM-1: Vascular cell adhesion molecule-1; VDAC: Voltage-dependent anion channel; VSMCs: Vascular smooth muscle cells. An upward arrow indicates an increase; a downward arrow indicates a decrease.

**Table 4:** Vaspin in vivo models of sepsis and organ injury.

Model	Species	Intervention	Key Findings	Mechanism	References
LPS-induced ARDS	Mouse	Ad-vaspin (systemic)	↓ Pulmonary inflammation; ↓ EC barrier dysfunction; preserved AJs; ↓ ICAM-1	Akt/GSK-3β activation	[33]
CLP-induced sepsis (cardiac)	Mouse	Recombinant vaspin pretreatment; *KLK7*-KO	↓ Mortality; ↓ CK-MB, LDH; ↓ CD45^+^/CD68^+^ cells; ↓ apoptosis; effects lost in KLK7-KO	KLK7 inhibition (serpin function)	[34]
Myocardial I/R	Mouse	AAV-vaspin (systemic)	↓ Infarct size; ↓ apoptosis; ↑ cardiac function; ↑ autophagic flux; chloroquine reversed	AMPK-mTOR → autophagic flux	[73]
Myocardial I/R	Rat	Vaspin (10–40 mg/kg)	↓ Infarct size (dose-dependent); ↓ CK-MB, LDH; ↓ IL-1β, IL-18, TNF-α	TLR4 ↓ → NF-κB ↓	[74]
Diabetic cardiomyopathy (STZ)	Rat	Vaspin (8 weeks IP)	↑ LVEF, FS; ↓ apoptosis; ↑ autophagy; ↓ NLRP3 inflammasome; improved mitochondria	Autophagy ↑ → NLRP3 ↓ (3-MA reversible)	[72,75]
Renal I/R injury	Mouse	Recombinant vaspin (SC)	↓ Tubular edema; ↓ netrin-1, L-FABP; ↓ inflammation; ↓ oxidative stress	HMGB1 ↓ → Nrf2/HO-1 ↑, NF-κB ↓	[76]
MI, TAC, Ang II infusion HF	Rat	vaspin (320-ng/kg/4 weeks/IP	↓ Fibrosis, ↓ hypertrophy	Suppresses PI3K/Akt; ↓ NADPH oxidase, ↓ superoxide, ↓ MDA	[80]
Carotid/femoral artery injury	Rat/Mouse	Ad-vaspin; Vaspin Tg mice	↓ Intimal proliferation; ↓ CCL2, PDGFRB expression	Endothelial protection; ↓ VSMC proliferation	[78]

Abbreviations: 3-MA: 3-Methyladenine; AAV-vaspin: Adeno-associated virus-vaspin; Ad-vaspin: Adenoviral-vaspin; AJ: Adherens junction; Akt: Protein kinase B; AMPK: Adenosine monophosphate-activated protein kinase; Ang II: Angiotensin II; ARDS: Acute respiratory distress syndrome; CCL2: C-C motif chemokine ligand 2; CD45: Cluster of differentiation 45; CD68: Cluster of differentiation 68; CK-MB: Creatine Kinase-MB; CLP: Cecal ligation and puncture; EC: Endothelial cell; FS: Fractional shortening; GSK-3β: Glycogen synthase kinase-3 beta; HF: Heart failure; HMGB1: High mobility group box 1; HO-1: Heme oxygenase-1; ICAM-1: Intercellular adhesion molecule-1; IP: Intraperitoneal; I/R: Ischemia/Reperfusion; KLK7: Kallikrein 7; KO: Knockout; LDH: Lactate Dehydrogenase; LPS: Lipopolysaccharide; LVEF: Left ventricular ejection fraction; L-FABP: Liver-type fatty acid-binding protein; MDA: Malondialdehyde; MI: Myocardial infarction; NF-κB: Nuclear factor-kappa B; NLRP3: NOD-, LRR- and pyrin domain-containing protein 3; Nrf2: Nuclear factor erythroid 2-related factor 2; PDGFRB: Platelet-derived growth factor receptor Beta; SC: Subcutaneous; STZ: Streptozotocin; TAC: Transverse aortic constriction; Tg: Transgenic; TLR4: Toll-like receptor 4; VSMCs: Vascular smooth muscle cells. An upward arrow indicates an increase; a downward arrow indicates a decrease.

**Table 5:** Omentin-1 in in vitro septic/inflammatory models.

Cell Type	Stimulus	Omentin-1 Effect	Mechanism	Key Outcome	References
U937 macrophages	LPS	Anti-inflammatory	TLR4/MyD88 ↓ → NF-κB ↓; Nrf2 ↑	↓ iNOS, COX-2, TNF-α, IL-6, IL-1β	[30]
RAW 264.7 macrophages	LPS	Anti-inflammatory	TXNIP ↓ → NLRP3 ↓	↓ Caspase-1, IL-1β, IL-18	[92]
Synovial fibroblasts → macrophages	Co-culture	M2 polarization	AMPK/PI3K/ERK/JAK → STAT6 → IL-4 ↑	↑ M2 markers; ↓ M1 markers	[87]
Human monocyte-derived macrophages	oxLDL	Anti-atherogenic, M2 shift	CD36/SR-A ↓; NCEH ↑	↓ Foam cell formation; M2 differentiation	[93]
HUVECs	TNF-α	Anti-inflammatory	AMPK → eNOS → NO → JNK ↓	↓ COX-2 (NO-dependent)	[94]
HUVECs	oxLDL	Anti-adhesive	p53 → KLF2 ↑ → eNOS ↑	↓ VCAM-1, E-selectin; ↓ THP-1 adhesion	[95]
HUVECs	Serum starvation	Pro-survival	AMPK → Akt → eNOS	↑ Tube formation; ↓ apoptosis	[88]
HPMECs	LPS (ARDS model)	Barrier-protective	Akt/eNOS	↑ VE-cadherin, F-actin; ↓ inflammation	[32]
Neonatal cardiomyocytes	H/R	Anti-apoptotic	AMPK + Akt (independent dual pathways)	↓ TUNEL, cleaved caspase-3	[96]
H9C2 cardiomyoblasts	Doxorubicin	Anti-apoptotic	Mitochondrial ROS ↓	↓ Caspase-3; ↓ MitoSOX	[97]
Cardiomyocytes	OGD	Mitochondrial protection	SIRT3/FOXO3a → fusion/fission balance, mitophagy	↑ Mfn2, OPA1; ↓ p-Drp1; ↑ PINK1/Parkin	[98]
Rat mesenteric VSMCs	PDGF-BB	Anti-migratory	NOX ↓ → ROS ↓ → p38/HSP27 ↓	↓ Migration (Boyden chamber)	[100]
VSMCs	Growth factors	Anti-proliferative	AMPK → ERK ↓	↓ Proliferation; ↓ neointimal formation in vivo	[99]
HASMCs	Ang II, PDGF-BB	Anti-atherogenic	Multiple	↓ Migration, proliferation, collagen expression	[93]
hPDLSCs → macrophages	LPS	Anti-inflammatory, M2 shift	ER stress ↓	↓ TNF-α, IL-1β, IL-6; ↑ M2 polarization	[101]

Abbreviations: ACAT-1: Acyl-CoA:cholesterol acyltransferase 1; AJ: Adherens junction; AMPK: Adenosine monophosphate-activated protein kinase; Ang II: Angiotensin II; CD36: Cluster of differentiation 36; COX-2: Cyclooxygenase-2; Drp1: Dynamin-related protein 1; eNOS: Endothelial nitric oxide synthase; ER: Endoplasmic reticulum; ERK: Extracellular signal-regulated kinase; F-actin: Filamentous actin; FOXO3a: Forkhead box protein O3a; H9C2: Rat cardiomyoblast cell line; HASMCs: Human aortic smooth muscle cells; hPDLSCs: Human periodontal ligament stem cells; HPMECs: Human pulmonary microvascular endothelial cells; HSP27: Heat shock protein 27; HUVECs: Human umbilical vein endothelial cells; H/R: Hypoxia/Reoxygenation; IL-1β/IL-4/IL-6/IL-18: Interleukin family; iNOS: Inducible nitric oxide synthase; JAK: Janus kinase; JNK: c-Jun N-terminal kinase; KLF2: Krüppel-like factor 2; LPS: Lipopolysaccharide; M2: Anti-inflammatory macrophage; Mfn2: Mitofusin-2; MyD88: Myeloid differentiation primary response 88; NCEH: Neutral cholesterol ester hydrolase; NF-κB: Nuclear factor-kappa B; NLRP3: NOD-, LRR- and pyrin domain-containing protein 3; NO: Nitric oxide; NOX: NADPH oxidase; Nrf2: Nuclear factor erythroid 2-related factor 2; OGD: Oxygen-glucose deprivation; OPA1: Optic atrophy 1; oxLDL: Oxidized low-density lipoprotein; p53: Protein 53; PDGF-BB: Platelet-derived growth factor-BB; PI3K/Akt: Phosphoinositide 3-kinase/Protein kinase B; PINK1/Parkin: PTEN-induced kinase 1/Parkin; RAW 264.7: Macrophage cell line; ROS: Reactive oxygen species; SIRT3: Sirtuin 3; SR-A: Scavenger receptor A; STAT6: Signal transducer and activator of transcription 6; THP-1: Human monocytic cell line; TLR4: Toll-like receptor 4; TXNIP: Thioredoxin-interacting protein; U937: Macrophage-like cell line; VCAM-1: Vascular cell adhesion molecule-1; VE-cadherin: Vascular endothelial cadherin; VSMCs: Vascular smooth muscle cells. An upward arrow indicates an increase; a downward arrow indicates a decrease.

**Table 6:** Omentin-1 in vivo models of sepsis and organ injury.

Model	Species	Intervention	Key Findings	Mechanism	References
LPS-induced ARDS (prophylactic)	Mouse	Ad-omentin (3d pre-LPS)	↓ Pulmonary inflammation; ↓ EC barrier injury; restored AJs/F-actin	Akt/eNOS activation	[32]
LPS-induced ARDS (therapeutic)	Mouse	rh-omentin (post-LPS)	Effective protection against established ARDS	Akt/eNOS activation	[32]
BLM-induced ALI	Mouse	Ad-omentin-1	↓ Lung injury; preserved alveolar septa; ↓ neutrophils, macrophages; ↓ MCP-1, IL-1β	NF-κB ↓; oxidative stress ↓	[102]
BLM-induced lung fibrosis	Mouse	Omentin-1	Reversed established fibrosis; myofibroblast → lipofibroblast reprogramming	PKM2/YAP ↓ → PPARγ ↑ → PLIN2 ↑	[103]
Myocardial I/R	Mouse	Ad-omentin; rh-omentin (0.1 μg/g IV)	↓ Infarct size; ↑ eNOS; ↓ NF-κB; 1-shot rh-omentin also effective	AMPK + Akt (independent dual pathways)	[96]
Myocardial ischemia-induced HF	Mouse	Fat-specific AAV-omentin1	↑ Cardiac function; ↓ hypertrophy; ↑ mitochondrial fusion; ↑ mitophagy	SIRT3/FOXO3a → Mfn2/OPA1 ↑, Drp1 ↓, PINK1/Parkin ↑	[98]
Hindlimb ischemia	Mouse	Ad-omentin; *eNOS*-KO	↑ Blood flow recovery, capillary density in WT; NO effect in *eNOS*-KO	AMPK → Akt → eNOS (essential)	[88]
Cerebral ischemia (MCAO)	Rat	LV-intelectin-1 (7d pre-MCAO)	↓ Infarct volume; ↑ CD34, capillary density; ↑ Bcl-2	Akt → eNOS	[104]
DSS-induced colitis	Mouse	rh-omentin-1 (IP)	↓ Inflammation; ↑ intestinal barrier; ↓ ROS/MDA; ↑ GSH/SOD	Nrf2 activation → NF-κB ↓	[105]
Collagen-induced arthritis	Mouse	Intra-articular omentin-1	↓ Arthritis; ↑ IL-4; ↑ M2 macrophages	AMPK/PI3K/ERK/JAK → STAT6 → IL-4	[87]
Atherosclerosis	*APOE*^−/−^ mouse	Omentin-1 infusion (4 weeks)	↓ Aortic lesions; ↓ macrophage/SMC content; ↓ inflammasome	M2 polarization; ↓ foam cells	[93]
Arterial wire injury	Mouse	Fat-specific omentin Tg	↓ Neointimal thickening; ↑ AMPK in injured arteries	AMPK → ERK ↓	[99]
Hemodynamic effects	Rat	Omentin-1 (8 μg/kg IP × 14d)	↓ MBP, PP; ↑ L-citrulline; ↑ adiponectin; ↓ IL-6 in PAT	NO-dependent vasodilation	[106]

Abbreviations: AAV-omentin1: Adeno-associated virus-omentin1; Ad-omentin: Adenoviral-omentin; AJ: Adherens junction; ALI: Acute lung injury; AMPK: Adenosine Monophosphate-activated protein kinase; ApoE: Apolipoprotein E; ARDS: Acute respiratory distress syndrome; Bcl-2: B-cell lymphoma 2; BLM: Bleomycin; Drp1: Dynamin-related protein 1; DSS: Dextran sulfate sodium; EC: Endothelial cell; eNOS: Endothelial nitric oxide synthase; eNOS: Endothelial nitric oxide synthase; F-actin: Filamentous actin; FOXO3a: Forkhead box protein O3a; GSH: Glutathione; HF: Heart failure; IL-1β/IL-4/IL-6: Interleukin family; IP: Intraperitoneal; I/R: Ischemia/Reperfusion; KO: Knockout; LPS: Lipopolysaccharide; LV: Lentiviral; MBP: Mean blood pressure; MCAO: Middle cerebral artery occlusion; MCP-1: Monocyte chemoattractant protein-1; MDA: Malondialdehyde; Mfn2: Mitofusin-2; NF-κB: Nuclear factor-kappa B; NO: Nitric oxide; Nrf2: Nuclear factor erythroid 2-related factor 2; OPA1: Optic atrophy 1; PAT: Periadventitial adipose tissue; PINK1/Parkin: PTEN-induced kinase 1/Parkin; PKM2: Pyruvate kinase M2; PLIN2: Perilipin 2; PP: Pulse pressure; PPARγ: Peroxisome proliferator-activated receptor gamma; rh-omentin: Recombinant human omentin; ROS: Reactive oxygen species; SIRT3: Sirtuin 3; SMC: Smooth muscle cell; SOD: Superoxide dismutase; Tg: Transgenic; WT: Wild-type; YAP: Yes-associated protein. An upward arrow indicates an increase; a downward arrow indicates a decrease.

**Table 7:** Integrated signaling pathway summary for chemerin, vaspin, and omentin-1 in the context of sepsis and organ injury.

Adipokine	Receptor/Target	Signaling Cascade	Net Effect on NF-κB	Organ Outcome	References
Chemerin	CMKLR1 (Gαi-coupled)	Gαi → ERK1/2 MAPK + PI3K/Akt → NF-κB ↑; adhesion molecule upregulation	Activation ↑	Endothelial inflammation; immune cell recruitment	[40,41,42]
Chemerin (resolution)	CMKLR1 (via RvE1/chemerin9)	Gi → pro-resolving macrophage signaling; pDC recruitment; GRK6/β-arr2 desensitization	Suppression ↓	Inflammation resolution (context-dependent)	[35,43]
Vaspin	KLK7 (serpin inhibition)	KLK7 inhibition → reduced cardiac inflammation; DNA binding accelerates inhibition 5-fold	Indirect ↓	Cardiac protection in CLP sepsis	[34,66,67]
Vaspin	AMPK activation	AMPK → IKKα/β ↓ → IκB ↓ → NF-κB ↓; adhesion molecule suppression; adipocyte IL-6/MCP-1/TNF-α ↓	Suppression ↓	Endothelial & adipose protection	[28,29]
Vaspin	Akt/GSK-3β pathway	Akt/GSK-3β → NF-κB ↓, apoptosis ↓, ROS ↓; via Akt/mTOR → autophagy ↑ (LC3-II, Beclin-1)	Suppression ↓	Lung (ARDS) + cardiac (remodeling) protection	[33,72,73,74,80]
Omentin-1	Akt/eNOS	Akt → eNOS → NO ↑ → VE-cadherin/F-actin restoration; endothelial barrier repair	Indirect ↓	Endothelial barrier in ARDS	[32,88]
Omentin-1	TLR4/MyD88 suppression	TLR4/MyD88 ↓ → p65 nuclear accumulation ↓ → iNOS/COX-2 ↓; Nrf2 nuclear translocation → HO-1/NQO1 ↑	Suppression ↓	Macrophage deactivation; antioxidant defense	[30,94,101,105]
Omentin-1	AMPK/PPARδ; Wnt5a/Ca^2+^	AMPK → PPARδ ↑ → ER stress ↓, ROS ↓; M2 polarization via STAT6/IL-4; Wnt5a/Ca^2+^ ↓ → mitochondrial biogenesis ↑	Indirect ↓	Endothelial dysfunction reversal; cardiac protection	[87,89,90]

Abbreviations: AJ: Adherens junction; AMPK: Adenosine monophosphate-activated protein kinase; ARDS: Acute respiratory distress syndrome; β-arr2: Beta-arrestin 2; CLP: Cecal ligation and puncture; CMKLR1: Chemokine-like receptor 1; COX-2: Cyclooxygenase-2; eNOS: Endothelial nitric oxide synthase; ER: Endoplasmic reticulum; ERK1/2: Extracellular signal-regulated kinase 1/2; Gαi: G-protein subunit alpha i; GRK6: G protein-coupled receptor kinase 6; GSK-3β: Glycogen synthase kinase-3 beta; HO-1: Heme oxygenase-1; IκB: Inhibitor of kappa B; IKKα/β: IkappaB kinase alpha/beta; IL-4: Interleukin-4; IL-6: Interleukin-6; iNOS: Inducible nitric oxide synthase; KLK7: Kallikrein 7; LC3-II: Microtubule-associated protein 1A/1B-light chain 3; MAPK: Mitogen-activated protein kinase; MCP-1: Monocyte chemoattractant protein-1; mTOR: Mammalian target of rapamycin; MyD88: Myeloid differentiation primary response 88; NF-κB: Nuclear factor-kappa B; NO: Nitric oxide; NQO1: NAD(P)H quinone dehydrogenase 1; Nrf2: Nuclear factor erythroid 2-related factor 2; p65: NF-κB subunit; pDC: Plasmacytoid dendritic cell; PI3K/Akt: Phosphoinositide 3-kinase/Protein kinase B; PPARδ: Peroxisome proliferator-activated receptor delta; ROS: Reactive oxygen species; RvE1: Resolvin E1; STAT6: Signal transducer and activator of transcription 6; TLR4: Toll-like receptor 4; TNF-α: Tumor necrosis factor-alpha; Wnt5a: Wnt family member 5A. An upward arrow indicates an increase; a downward arrow indicates a decrease.

**Table 8:** Comparative summary of the findings in septic/inflammatory/ALI animal models.

Adipokine	Model Tested	Net In Vivo Effect	Lung Protection	References
Chemerin	LPS-ALI; zymosan/thioglycollate peritonitis; peritoneal sepsis	Anti-inflammatory (via ChemR23); pro-inflammatory when unregulated (*CCRL2*-KO)	↓ Neutrophil infiltration; ↓ cytokines (ChemR23-dependent)	[47,49,52,53,54,60]
Vaspin	LPS-ARDS; CLP sepsis (cardiac)	Anti-inflammatory; cardioprotective	↓ EC barrier dysfunction; ↓	[33,34]
Omentin-1	LPS-ARDS (prophylactic + therapeutic); BLM-ALI	Uniformly anti-inflammatory; organ-protective inflammation; preserved AJs	↓ Inflammation; restored AJs/F-actin; ↓ barrier permeability; reversed fibrosis	[32,102]

Abbreviations: AJ: Adherens junction; ALI: Acute lung injury; ARDS: Acute respiratory distress syndrome; BLM: Bleomycin; CCRL2: C-C motif chemokine receptor-Like 2; ChemR23: Chemerin receptor 23; CLP: Cecal ligation and puncture; EC: Endothelial cell; F-actin: Filamentous actin; KO: Knockout; LPS: Lipopolysaccharide. An upward arrow indicates an increase; a downward arrow indicates a decrease.

**Table 9:** Comparative summary of the clinical evidence in sepsis/critical illness.

Feature	Chemerin	Vaspin	Omentin-1	References
Largest sepsis clinical study	Karampela: *n* = 102 septic ICU (prospective)	Motal: *n* = 57 septic ICU (prospective)	Karampela: *n* = 102 septic ICU (prospective); Luedde: *n* = 117 ICU (84 septic)	[21,23,83,84]
Change in sepsis	Elevated (1.7-fold vs. controls)	Elevated (3-fold vs. ICU controls	Elevated (1.69-fold vs. controls, Karampela); unchanged (Luedde); lower in ARDS (Qi)	[21,23,32,60,62,83,84]
Sepsis diagnostic performance	AUC: 0.78 (comparable to CRP)	Not studied	AUC > 0.739 (comparable to CRP)	[21,23]
Septic shock vs. sepsis	Higher	Not studied	Higher	[21,23]
28-day mortality HR (onset)	3.58 (95% CI 1.48–8.65, *p* = 0.005)	Not studied	2.26 (95% CI 1.21–4.19, *p* = 0.01)	[21,23]
28-day mortality HR (1 week)	10.01 (95% CI 4.32–23.20, *p* 0.001)	Not studied	2.15 (95% CI 1.43–3.22, *p* < 0.001)	[21,23]
Non-survivors vs. survivors	Higher	Not studied	Higher	[21,23]
Kinetic trajectory (survivors)	Significant decline from Day 1 to Day 7	Not studied	Progressive physiological clearance	[21,23]
Kinetic trajectory (non-survivors)	Sustained elevation; failure to clear	Not studied	Smaller decline; sustained elevation)	[21,23]
Correlation with severity scores	SOFA, APACHE II, lactate, CRP, PCT	CRP, SAPS II, SOFA	SOFA, APACHE II	[21,83,84]
Long-term survival (>28 days)	Not studied	Not studied	Low admission levels favor long-term survival	[84]
Glucose metabolism link	Correlates with glucose, HOMA-IR; context-dependent mortality (SHG vs. non-SHG)	Negatively associated with CRP in hemodialysis	Not studied	[19,64]
Adipocytokine network	Clusters with metabolic adipokines, not the core inflammatory network	Not characterized	Correlates with leptin receptor, adiponectin (Luedde)	[84]

Abbreviations: APACHE II: Acute physiology and chronic health evaluation II; ARDS: Acute respiratory distress syndrome; AUC: Area under the curve; CI: Confidence interval; CRP: C-reactive protein; HOMA-IR: Homeostatic model assessment for insulin resistance; HR: Hazard ratio; ICU: Intensive care unit; PCT: Procalcitonin; SAPS II, Simplified acute physiology score II; SHG: Stress hyperglycemia; SOFA: Sequential organ failure assessment.

**Table 10:** Comparative summary of the COVID-19 studies.

Study	*n*	Chemerin	Vaspin	Omentin	Severity Correlation	References
Kukla et al. 2021	70 COVID + 20 HC	Lower	No change	Lower	None for any adipokine	[19]
Lavis et al. 2022	88 COVID (40 ICU) + 21 HC	Higher in ICU; higher in deceased; independent mortality predictor at day 14	Not measured	Not measured	Chemerin correlated with CRP, TNF-α	[62]
Wikar et al. 2026	40 COVID + 24 HC	Higher	Not measured	No change	Chemerin only; omentin not associated	[63]

Abbreviations: COVID-19: Coronavirus disease 2019; CRP: C-reactive protein; HC: Healthy control; ICU: Intensive care unit; TNF-α: Tumor necrosis factor-alpha.

## Data Availability

No new data were created or analyzed in this study. Data sharing is not applicable to this article.

## Supplementary Materials

The following supporting information can be downloaded at https://www.mdpi.com/article/10.3390/biomedicines14071553/s1, Table S1: Comparative summary of levels and functions of chemerin, vaspin, and omentin-1 from experimental evidence and clinical biomarker data in sepsis and critical illness; Table S2: The broader adipokine network in sepsis; Table S3: The complete list of abbreviations. References in supplementary materials file can be found [10,14,16,17,18,19,21,23,30,32,33,34,36,74,83,84,112,113,120].

## Abbreviations

The abbreviations used in this manuscript are listed in Table S3.
