# Supplementary material for: Novel Adipokines in Critical Illness and Sepsis: Chemerin, Vaspin, and Omentin-1: A Comprehensive Evidence-Based Review
Source: Biomedicines. 2026 Jul 10;14(7):1553. doi: 10.3390/biomedicines14071553 (PMC13405776; doi:10.3390/biomedicines14071553)
Supplement: Supplementary file 1 [file biomedicines-14-01553-s001.zip › biomedicines-4404359-supplementary.pdf]

Supplementary Table S1. Comparative summary of levels and functions of chemerin, vaspin, and omentin-1 from experimental evidence and clinical biomarker data in sepsis and critical illness.

Adipokine	Level in Sepsis	Key Functions	Severity Correlation	28-Day Mortality
**Chemerin**	Elevated [1]; no change [2]	Chemoattraction; antimicrobial; pro-resolving via CMKLR1; metabolic regulation [1,3]	APACHE II, SOFA, lactate, CRP, PCT [1]	Yes, HR 3.58 (onset); HR 10.01 (week 1) [1]
**Vaspin (*SERPINA12*)**	Elevated [4]	Serine protease inhibitor; anti-inflammatory via KLK7/AMPK/NF-κB; cardioprotective [5-7]	CRP, SAPS II [4]	Exploratory data only [4]
**Omentin-1 (Intelectin-1)**	Elevated [8]; unchanged in broader ICU cohort [9]	NF-κB/TLR4 suppression; M2 macrophage polarization; endothelial & pulmonary protection [10,11]	APACHE II, SOFA, coagulation [8]	Yes, HR 2.26 (onset); HR 2.15 (week 1) [8]

Abbreviations: AMPK: Adenosine monophosphate-activated protein kinase; APACHE II: Acute physiology and chronic health evaluation II; CRP: C-reactive protein; HR: Hazard ratio; KLK7: Kallikrein 7; M2: Anti-inflammatory macrophage polarization; NF-κB: Nuclear factor-kappa B; PCT: Procalcitonin; SERPINA12: Serine protease inhibitor A12; SOFA: Sequential organ failure assessment; TLR4: Toll-like receptor 4.

Supplementary Table S2. The broader adipokine network in sepsis.

Adipokine	Direction in Sepsis	Primary Role	Network Integration	Prognostic Value	References
Resistin	↑↑↑ (6-fold)	Pro-inflammatory	Core cluster with IL-6, IL-8, MCP-1, IL-10	Strong; predicts 28-day mortality	[12-14]
Chemerin	↑↑ (context-dependent)	Chemoattractant/ metabolic	Parallel pathway, not in core cluster	Moderate; predicts mortality, context-dependent	[1,13]
Visfatin/eNampt	↑↑	Pro-inflammatory	Correlates with resistin; joint sustained elevation predicts mortality	Strong; sustained elevation predicts mortality	[12,15]
Leptin	Variable (BMI-dependent)	Pro-inflammatory	BMI-driven, not endotype-associated	Weak/inconsistent	[12,16]}
Adiponectin	↓ acutely → ↑ recovery	Anti-inflammatory/protective	Reciprocal to resistin	Complex; gradual rise suggests recovery	[17,18]
Vaspin	↑ (exploratory data)	Anti-inflammatory/organ-protective	Independent pathway (KLK7, AMPK)	Under investigation	[4,13]
Omentin-1	↓ in acute illness (Direction differs across studies and patient populations (sepsis vs. ARDS vs. general ICU).	Anti-inflammatory/endothelial barrier	Independent pathway (Akt/eNOS, TLR4)	Paradoxical; low levels predict better long-term survival	[10,19]

Abbreviations: Akt: Protein kinase B; AMPK: Adenosine monophosphate-activated protein kinase; BMI: Body mass index; eNampt: Extracellular nicotinamide phosphoribosyltransferase; eNOS: Endothelial nitric oxide synthase; IL-6/IL-8/IL-10: Interleukin-6/Interleukin-8/Interleukin-10; KLK7: Kallikrein 7; MCP-1: Monocyte chemoattractant protein-1; TLR4: Toll-like receptor 4. An upward arrow indicates an increase; a downward arrow indicates a decrease.

Supplementary Table S3. The complete list of abbreviations.

Abbreviation	Full Term
3-MA	3-Methyladenine
3T3-L1	Murine pre-adipocyte cell line
AAV	Adeno-Associated Virus
ACAT-1	Acyl-CoA:Cholesterol Acyltransferase 1
ACCP/SCCM	American College of Chest Physicians/Society of Critical Care Medicine
Ad	Adenoviral (vector)
ADMA	Asymmetric Dimethylarginine
AdipoR	Adiponectin Receptor
AJ	Adherens Junction
AKI	Acute Kidney Injury
Akt	Protein Kinase B
ALI	Acute Lung Injury
AMPK	Adenosine Monophosphate-Activated Protein Kinase (also 5' AMP-activated protein kinase)
Ang II	Angiotensin II
APACHE II	Acute Physiology and Chronic Health Evaluation II
APOE	Apolipoprotein E
ARE	Antioxidant Response Element
ARDS	Acute Respiratory Distress Syndrome
ATF6	Activating Transcription Factor 6
AUC	Area Under the Curve
BALF	Bronchoalveolar Lavage Fluid
Bcl-2	B-Cell Lymphoma 2
BiP	Binding Immunoglobulin Protein (see GRP78)

Abbreviation	Full Term
BLM	Bleomycin
BLT1	Leukotriene B4 Receptor 1
BMI	Body Mass Index
C15	Chemerin15 peptide
cAMP	Cyclic Adenosine Monophosphate
CCL2	C-C Motif Chemokine Ligand 2
CCRL2	C-C Motif Chemokine Receptor-Like 2
CD34	Cluster of Differentiation 34
CD36	Cluster of Differentiation 36
CD45	Cluster of Differentiation 45
CD68	Cluster of Differentiation 68
cGMP	Cyclic Guanosine Monophosphate
ChemR23	Chemerin Receptor 23 (see CMKLR1)
CHOP	CCAAT-Enhancer-Binding Protein Homologous Protein
CI	Confidence Interval
CK-MB	Creatine Kinase-MB isoform
CLP	Cecal Ligation and Puncture
CMKLR1	Chemokine-Like Receptor 1 (Chemerin Receptor)
COX-2	Cyclooxygenase-2
COVID-19	Coronavirus Disease 2019
CRP	C-Reactive Protein
cryo-EM	Cryo-Electron Microscopy
CT	Computed Tomography
CXCL1	C-X-C Motif Chemokine Ligand 1
DAMP	Damage-Associated Molecular Pattern

Abbreviation	Full Term
DC	Dendritic Cell
DIC	Disseminated Intravascular Coagulation
Drp1	Dynamin-Related Protein 1
DSS	Dextran Sulfate Sodium
E. coli	Escherichia coli
EA.hy926	Human hybrid endothelial cell line
EC	Endothelial Cell
ECG	Electrocardiogram
eNampt	Extracellular Nicotinamide Phosphoribosyltransferase
ENaC	Epithelial Sodium Channel
eNOS	Endothelial Nitric Oxide Synthase
ER	Endoplasmic Reticulum
ERK1/2	Extracellular Signal-Regulated Kinase 1/2
F-actin	Filamentous Actin
FGF-21	Fibroblast Growth Factor 21
FOXO3a	Forkhead Box Protein O3a
FS	Fractional Shortening
Gαi	G-Protein Subunit Alpha i
GGT	Gamma-Glutamyl Transferase
GLP-1	Glucagon-Like Peptide-1
GPCR	G Protein-Coupled Receptor
GPR1	G Protein-Coupled Receptor 1
GRK6	G Protein-Coupled Receptor Kinase 6
GRP78	Glucose-Regulated Protein 78 kDa (also BiP)
GSH	Glutathione

Abbreviation	Full Term
GSK-3β	Glycogen Synthase Kinase-3 Beta
H/R	Hypoxia/Reoxygenation
H9C2	Rat cardiomyoblast cell line
HAECs	Human Aortic Endothelial Cells
HASMCs	Human Aortic Smooth Muscle Cells
HC	Healthy Control
HF	Heart Failure
HFpEF	Heart Failure with Preserved Ejection Fraction
HMGB1	High Mobility Group Box 1
HMVECs	Human Microvascular Endothelial Cells
HO-1	Heme Oxygenase-1
HOMA-IR	Homeostatic Model Assessment for Insulin Resistance
hPDLSCs	Human Periodontal Ligament Stem Cells
HPMECs	Human Pulmonary Microvascular Endothelial Cells
HR	Hazard Ratio
HSP27	Heat Shock Protein 27
HSPA5	Gene encoding GRP78/BiP
HUVECs	Human Umbilical Vein Endothelial Cells
ICAM-1	Intercellular Adhesion Molecule-1
ICU	Intensive Care Unit
IFN-γ	Interferon-Gamma
IGF-1	Insulin-Like Growth Factor-1
IKK / IKKα/β	IκB Kinase / IκB Kinase Alpha/Beta
IL-1 / IL-1β	Interleukin-1 / Interleukin-1 Beta
IL-10	Interleukin-10

Abbreviation	Full Term
IL-18	Interleukin-18
IL-4	Interleukin-4
IL-6	Interleukin-6
IL-8	Interleukin-8
iNOS	Inducible Nitric Oxide Synthase
IP	Intraperitoneal
I/R	Ischemia/Reperfusion
IκB	Inhibitor of Kappa B
IV	Intravenous
JAK	Janus Kinase
JNK	c-Jun N-Terminal Kinase
Kd	Dissociation Constant
KLF2	Krüppel-Like Factor 2
KLK7	Kallikrein 7
KO	Knockout
L-FABP	Liver-Type Fatty Acid-Binding Protein
LC3-II/I	Microtubule-Associated Protein 1A/1B-Light Chain 3
LDH	Lactate Dehydrogenase
LPS	Lipopolysaccharide
LRP1	Low-Density Lipoprotein Receptor-Related Protein 1
LV	Lentiviral (vector)
LVEF	Left Ventricular Ejection Fraction
M1 / M2	Pro-inflammatory / Anti-inflammatory Macrophage Phenotype
MAPK	Mitogen-Activated Protein Kinase
MBP	Mean Blood Pressure

Abbreviation	Full Term
MCAO	Middle Cerebral Artery Occlusion
MCP-1	Monocyte Chemoattractant Protein-1
MDA	Malondialdehyde
Mfn2	Mitofusin-2
MI	Myocardial Infarction
MODS	Multiple Organ Dysfunction Syndrome
MPO	Myeloperoxidase
MQTiPSS	Minimum Quality Threshold in Pre-Clinical Sepsis Studies
mTOR	Mammalian Target of Rapamycin
MTJ-1	Murine DnaJ Homolog 1
MyD88	Myeloid Differentiation Primary Response 88
NAC	N-Acetylcysteine
NADPH	Nicotinamide Adenine Dinucleotide Phosphate
NCEH1	Neutral Cholesterol Ester Hydrolase 1
NF-κB	Nuclear Factor Kappa-Light-Chain-Enhancer of Activated B Cells
NK	Natural Killer (cell)
NKA	Na+/K+-ATPase
NLRP3	NOD-, LRR- and Pyrin Domain-Containing Protein 3
NO	Nitric Oxide
NOX	NADPH Oxidase
NQO1	NAD(P)H Quinone Dehydrogenase 1
Nrf2	Nuclear Factor Erythroid 2-Related Factor 2
OGD	Oxygen-Glucose Deprivation
OPA1	Optic Atrophy 1
oxLDL	Oxidized Low-Density Lipoprotein

Abbreviation	Full Term
p38	p38 Mitogen-Activated Protein Kinase
p53	Protein 53 (tumor suppressor)
p65	NF-κB subunit p65
PAI-1	Plasminogen Activator Inhibitor-1
PAT	Periadventitial Adipose Tissue
PCT	Procalcitonin
pDC	Plasmacytoid Dendritic Cell
PDGF-BB / PDGFB	Platelet-Derived Growth Factor-BB / Subunit B
PDGFRB	Platelet-Derived Growth Factor Receptor Beta
PI3K	Phosphoinositide 3-Kinase
PINK1/Parkin	PTEN-Induced Kinase 1 / Parkin (mitophagy pathway)
PKCθ	Protein Kinase C Theta
PKM2	Pyruvate Kinase M2
PLIN2	Perilipin 2
PP	Pulse Pressure
PPARδ	Peroxisome Proliferator-Activated Receptor Delta
PPARγ	Peroxisome Proliferator-Activated Receptor Gamma
PVM	Pneumonia Virus of Mice
RARRES2	Retinoic Acid Receptor Responder 2 (gene encoding chemerin)
RAS	Renin-Angiotensin System
RAW 264.7	Murine macrophage cell line
rh-omentin	Recombinant Human Omentin
rM-ed	Reverse-Migrated (neutrophils)
ROS	Reactive Oxygen Species
RvE1	Resolvin E1

Abbreviation	Full Term
SAA	Serum Amyloid A
SAPS II	Simplified Acute Physiology Score II
SARS-CoV-2	Severe Acute Respiratory Syndrome Coronavirus 2
SAT	Subcutaneous Adipose Tissue
SC	Subcutaneous
scRNA-seq	Single-Cell RNA Sequencing
SERPINA12	Serine Protease Inhibitor A12 (gene encoding vaspin)
SGK1	Serum/Glucocorticoid-Regulated Kinase 1
SHG	Stress Hyperglycemia
siRNA	Small Interfering RNA
SIRT3	Sirtuin 3
SMC	Smooth Muscle Cell
SOD	Superoxide Dismutase
SOFA	Sequential Organ Failure Assessment
SR-A	Scavenger Receptor A
STAT6	Signal Transducer and Activator of Transcription 6
STZ	Streptozotocin
Syk	Spleen Tyrosine Kinase
TAC	Transverse Aortic Constriction
TGF-β	Transforming Growth Factor Beta
THP-1	Human monocytic cell line
TLR4	Toll-Like Receptor 4
TNF-α	Tumor Necrosis Factor-Alpha
TUNEL	Terminal Deoxynucleotidyl Transferase dUTP Nick End Labeling
TXNIP	Thioredoxin-Interacting Protein

Abbreviation	Full Term
U937	Human macrophage-like cell line
VAT	Visceral Adipose Tissue
VCAM-1	Vascular Cell Adhesion Molecule-1
VDAC	Voltage-Dependent Anion Channel
VE-cadherin	Vascular Endothelial Cadherin
VLA-4 / VLA-5	Very Late Antigen-4 / Very Late Antigen-5 (integrins α4β1 / α5β1)
VSMCs	Vascular Smooth Muscle Cells
WAT	White Adipose Tissue
WBC	White Blood Cell
Wnt5a	Wnt Family Member 5A
WT	Wild-Type
YAP	Yes-Associated Protein
β-arrestin 2	Beta-Arrestin 2

References

1. Karampela, I.; Christodoulatos, G.S.; Vallianou, N.; Tsilingiris, D.; Chrysanthopoulou, E.; Skyllas, G.; Antonakos, G.; Marinou, I.; Vogiatzakis, E.; Armaganidis, A.; et al. Circulating Chemerin and Its Kinetics May Be a Useful Diagnostic and Prognostic Biomarker in Critically Ill Patients with Sepsis: A Prospective Study. *Biomolecules* **2022**, *12*, 301, doi:10.3390/biom12020301.
2. Kukla, M.; Menżyk, T.; Dembiński, M.; Winiarski, M.; Garlicki, A.; Bociąga-Jasik, M.; Skonieczna, M.; Hudy, D.; Maziarz, B.; Kusnierz-Cabala, B.; et al. Anti-inflammatory adipokines: chemerin, vaspin, omentin concentrations and SARS-CoV-2 outcomes. *Scientific reports* **2021**, *11*, 21514, doi:10.1038/s41598-021-00928-w.
3. Bondue, B.; Wittamer, V.; Parmentier, M. Chemerin and Its Receptors in Leukocyte Trafficking, Inflammation and Metabolism. *Cytokine Growth Factor Rev* **2011**, *22*, 331-338, doi:10.1016/j.cytogfr.2011.11.004.
4. Motal, M.C.; Klaus, D.A.; Lebherz-Eichinger, D.; Tudor, B.; Hamp, T.; Wiegele, M.; Seemann, R.; Krenn, C.G.; Roth, G.A. Increased plasma vaspin concentration in patients with sepsis: an exploratory examination. *Biochem Med (Zagreb)* **2015**, *25*, 90-96, doi:10.11613/bm.2015.011.
5. Qi, D.; Wang, D.; Zhang, C.; Tang, X.; He, J.; Zhao, Y.; Du, J. Vaspin Protects Against LPS-Induced ARDS by Inhibiting Inflammation, Apoptosis and Reactive Oxygen Species Generation in Pulmonary Endothelial Cells via the Akt/GSK-3beta Pathway. *Int J Mol Med* **2017**, *40*, 1803-1817, doi:10.3892/ijmm.2017.3176.
6. Yin, N.; Pan, F.; Qiu, L.; Yang, Z.; Xiong, R.; Shi, L.; Shi, Y.; Wu, N.; Wu, K.; Li, Q.; et al. Vaspin Alleviates Sepsis-Induced Cardiac Injury and Cardiac Inflammation by Inhibiting Kallikrein 7 in Mice. *Mediators Inflamm* **2022**, *2022*, 1149582, doi:10.1155/2022/1149582.

7. Yuan, L.; Dai, X.; Fu, H.; Sui, D.; Lin, L.; Yang, L.; Zha, P.; Wang, X.; Gong, G. Vaspin protects rats against myocardial ischemia/reperfusion injury (MIRI) through the TLR4/NF-κB signaling pathway. *Eur J Pharmacol* **2018**, *835*, 132-139, doi:10.1016/j.ejphar.2018.07.052.
8. Karampela, I.; Vallianou, N.G.; Tsilingiris, D.; Christodoulatos, G.S.; Antonakos, G.; Marinou, I.; Vogiatzakis, E.; Armaganidis, A.; Dalamaga, M. Diagnostic and Prognostic Value of Serum Omentin-1 in Sepsis: A Prospective Study in Critically Ill Patients. *Medicina (Kaunas)* **2023**, *59*, 833, doi:10.3390/medicina59050833.
9. Luedde, M.; Benz, F.; Niedeggen, J.; Vucur, M.; Hippe, H.J.; Spehlmann, M.E.; Schueller, F.; Loosen, S.; Frey, N.; Trautwein, C.; et al. Elevated Omentin Serum Levels Predict Long-Term Survival in Critically Ill Patients. *Dis Markers* **2016**, *2016*, 3149243, doi:10.1155/2016/3149243.
10. Qi, D.; Tang, X.; He, J.; Wang, D.; Zhao, Y.; Deng, W.; Du, J. Omentin Protects Against LPS-Induced ARDS Through Suppressing Pulmonary Inflammation and Promoting Endothelial Barrier via an Akt/eNOS-Dependent Mechanism. *Cell Death Dis* **2016**, *7*, e2360, doi:10.1038/cddis.2016.265.
11. Wang, J.; Gao, Y.; Lin, F.; Han, K.; Wang, X. Omentin-1 Attenuates LPS-Induced Macrophage Activation by Inhibiting TLR4/MyD88/NF-kappaB Signaling. *Arch Biochem Biophys* **2020**, *679*, 108196, doi:10.1016/j.abb.2019.108196.
12. de Nooijer, A.H.; Antonakos, N.; Markopoulou, D.; Grondman, I.; Pickkers, P.; Netea, M.G.; Giamarellos-Bourboulis, E.J. The Role of Obesity and Plasma Adipocytokines in Immune Dysregulation in Sepsis Patients. *Shock* **2023**, *59*, 214-222, doi:10.1097/shk.0000000000002046.
13. Du, Q.; He, F.; Zhang, S.; Lan, X.; Pan, Y.; Wang, J. Current understanding and advances regarding the adipose-immune-metabolic axis in disease tolerance during sepsis. *Frontiers in immunology* **2026**, *17*, 1755423, doi:10.3389/fimmu.2026.1755423.
14. Ebihara, T.; Matsumoto, H.; Matsubara, T.; Matsuura, H.; Hirose, T.; Shimizu, K.; Ogura, H.; Kang, S.; Tanaka, T.; Shimazu, T. Adipocytokine Profile Reveals Resistin Forming a Prognostic-Related Cytokine Network in the Acute Phase of Sepsis. *Shock* **2021**, *56*, 718-726, doi:10.1097/shk.0000000000001756.
15. Karampela, I.; Christodoulatos, G.S.; Kandri, E.; Vogiatzakis, E.; Yllou, E.; Hamodrakas, S.J.; Dalamaga, M. Circulating eNampt and Resistin as a Proinflammatory Duet Predicting Independently Mortality in Critically Ill Patients with Sepsis. *Cytokine* **2019**, *120*, 219-226, doi:10.1016/j.cyto.2019.04.006.
16. Karampela, I.; Chrysanthopoulou, E.; Skyllas, G.; Christodoulatos, G.S.; Kandri, E.; Antonakos, G.; Stratigou, T.; Armaganidis, A.; Dalamaga, M. Circulating leptin, soluble leptin receptor and free leptin index in critically ill patients with sepsis: a prospective observational study. *Minerva Anestesiol* **2021**, *87*, 880-890, doi:10.23736/s0375-9393.21.15368-4.
17. Gianoli, S.; Tang, J.; Odegard, K.C.; Yuki, K.; Koutsogiannaki, S. Harnessing Adiponectin for Sepsis: Current Knowledge, Clinical Insights and Future Therapies. *Crit Care* **2025**, *29*, 251, doi:10.1186/s13054-025-05478-9.
18. Vassiliadi, D.A.; Tzanela, M.; Kotanidou, A.; Orfanos, S.E.; Nikitas, N.; Armaganidis, A.; Tsagarakis, S. Serial Changes in Adiponectin and Resistin in Critically Ill Patients with Sepsis. *J Crit Care* **2012**, *27*, 316.e311-316.e319, doi:10.1016/j.jcrc.2011.07.080.
19. Alipoor, E.; Mohammad Hosseinzadeh, F.; Hosseinzadeh-Attar, M.J. Adipokines in critical illness: A review of the evidence and knowledge gaps. *Biomed Pharmacother* **2018**, *108*, 1739-1750, doi:10.1016/j.biopha.2018.09.165.
